# Re-awakening the brain: Forcing transitions in disorders of consciousness by external *in silico* perturbation

**DOI:** 10.1371/journal.pcbi.1011350

**Published:** 2024-05-03

**Authors:** Paulina Clara Dagnino, Anira Escrichs, Ane López-González, Olivia Gosseries, Jitka Annen, Yonatan Sanz Perl, Morten L. Kringelbach, Steven Laureys, Gustavo Deco

**Affiliations:** 1 Computational Neuroscience Group, Center for Brain and Cognition, Department of Information and Communication Technologies, Universitat Pompeu Fabra, Barcelona, Catalonia, Spain; 2 Coma Science Group, GIGA Consciousness, University of Liège, Liège, Belgium; 3 Centre du Cerveau 2, University Hospital of Liège, Liège, Belgium; 4 Institut du Cerveau et de la Moelle épinière, ICM, Paris, France; 5 Centre for Eudaimonia and Human Flourishing, University of Oxford, Oxford, United Kingdom; 6 Department of Psychiatry, University of Oxford, Oxford, United Kingdom; 7 Center for Music in the Brain, Department of Clinical Medicine, Aarhus University, Aarhus, Denmark; 8 Joint International Research Unit on Consciousness, CERVO Brain Research Centre, University of Laval, Québec, Québec, Canada; 9 Institució Catalana de la Recerca i Estudis Avançats (ICREA), Barcelona, Catalonia, Spain; Max Planck Institute for Biological Cybernetics, GERMANY

## Abstract

A fundamental challenge in neuroscience is accurately defining brain states and predicting how and where to perturb the brain to force a transition. Here, we investigated resting-state fMRI data of patients suffering from disorders of consciousness (DoC) after coma (minimally conscious and unresponsive wakefulness states) and healthy controls. We applied model-free and model-based approaches to help elucidate the underlying brain mechanisms of patients with DoC. The model-free approach allowed us to characterize brain states in DoC and healthy controls as a probabilistic metastable substate (PMS) space. The PMS of each group was defined by a repertoire of unique patterns (i.e., metastable substates) with different probabilities of occurrence. In the model-based approach, we adjusted the PMS of each DoC group to a causal whole-brain model. This allowed us to explore optimal strategies for promoting transitions by applying off-line *in silico* probing. Furthermore, this approach enabled us to evaluate the impact of local perturbations in terms of their global effects and sensitivity to stimulation, which is a model-based biomarker providing a deeper understanding of the mechanisms underlying DoC. Our results show that transitions were obtained in a synchronous protocol, in which the somatomotor network, thalamus, precuneus and insula were the most sensitive areas to perturbation. This motivates further work to continue understanding brain function and treatments of disorders of consciousness.

## Author summary

We studied disorders of consciousness (DoC) by defining a brain state as a repertoire of metastable substates with different probabilities of occurrence. We created whole-brain computational models of DoC to uncover the causal mechanisms underlying recovery. These models allowed us to simulate transitions by studying the effects of artificial individual local perturbations under different protocol regimes. We demonstrated successful transitions in the synchronization protocol and showed that the most sensitive areas were located in the somatomotor network, thalamus, precuneus and insula. In the long-term, this could bring valuable insights for understanding DoC and open new avenues for future clinical therapies.

## Introduction

The brain is a dynamical, complex, and self-organized system with spontaneous activity emerging from non-linear interactions of billions of neurons [1]. Current research is increasing our understanding of the causal dynamics underlying different brain states, such as wakefulness, sleep, anesthesia, and disorders of consciousness (DoC). Nevertheless, such mechanisms still remain elusive and a deeper comprehension would facilitate the design of novel treatments for brain disorders and possibly for the loss of consciousness like coma. Recently, directly perturbing the brain *in silico* has been proposed to investigate the dynamical mechanisms of brain states in health and disease [2–5]. Furthermore, such perturbations could be used to study the sensitivity of brain areas and identify model-based biomarkers.

A healthy brain relies on the brain’s flexibility and capacity to integrate information and maintain rich dynamics in an evolving environment across time and space [6]. By contrast, brain disorders present disruptions in the normal range of brain activity [7]. In the field of consciousness studies, DoC refers to patients after severe brain injuries (e.g., trauma, anoxia, stroke). In the clinical domain, post-coma states (i.e., DoC) are distinguished into the minimally conscious state (MCS) and unresponsive wakefulness syndrome (UWS) [8–11]. MCS is identified when patients are awake and respond with limited and fluctuant awareness, and UWS corresponds to patients with reflexive behavior who do not respond to stimulation consciously. Past research has revealed DoC patients present lower flexibility and efficiency of information processing and a limited broadcast of information, which coexists with a reduced neural propagation and responsiveness to events [12]. In addition, they present disruptions of long-range cortical correlations [13], loss of integration and increased segregation [14], and reduced brain dynamics and heterogeneity [15–18].

In recent years, different definitions of brain states have been proposed using empirical neuroimaging and electrophysiological data. Approaches based on functional magnetic resonance imaging (fMRI) have implemented static analysis such as long-range temporal dependence via Hurst exponent [19] and attractors between brain regions [20, 21]. Recognizing brain activity is multi-dimensional and ever-changing, these approaches have been further examined from a more realistic and richer viewpoint considering brain dynamics [22–27]. This time-resolved perspective aligns with the shared view of brain state as evolving dynamic patterns of brain activity emerging from and impacting physiology and/or behavior [28]. Nevertheless, a universal, formal, robust, and quantitative definition of brain state, and a deep comprehension of the effects of perturbations to force transitions, remains unknown [5]. Stemming from recent progress in these areas, we could still benefit from a better understanding of brain dynamics and optimal transition strategies between brain states [29–31].

There is a long tradition of perturbative approaches for brain research. Clinical techniques for stimulation exist, such as the non-invasive transcranial direct current stimulation (tDCS) [32–34], transcranial magnetic stimulation (TMS) [35, 36], and deep brain stimulation (DBS) [37, 38]. Still, research in lesioned humans is rare, only undertaken when the disease is severe, and accompanied by ethical constraints [39, 40]. An approach used to distinguish brain states is the perturbational complexity index (PCI) developed by Massimini and colleagues. This method calculates the lempel-ziv complexity (i.e., compressibility in terms of repetitiveness of the signal sequence) from the electroencephalography response to TMS perturbation [41–43]. In other words, the PCI measures the perturbation-elicited variations in intrinsic global brain activity and has shown to be successful in distinguishing between awake vs. sleep, awake vs. anesthesia, and MCS vs. UWS [41–44]. Overall, given the ethical limitations of empirical neurostimulation approaches in non-communicative patients, causal whole-brain models based on *in silico* perturbation protocols are fundamental to understanding the underlying mechanisms of brain dynamics. This promising tool allows experimenting in unprecedented unlimited scenarios (e.g., perturbing one brain area at a time) without exposing real brains [6, 45, 46].

Recently, the awakening framework was proposed consisting of model-free and model-based approaches to force transitions from deep sleep to awake [5]. In particular, the model-free approach based on Leading Eigenvector Dynamics Analysis (LEiDA) [47] uses the concept of metastability, which refers to the characteristic of a system to maintain an equilibrium in a temporal window although being slightly perturbed [48–50]. Here, brain dynamics in a particular brain state are defined by a repertoire of metastable substates (i.e., dynamic patterns) around critical points between order and chaos [47, 51]. In other words, the nature, duration, and arrangement of existent metastable substates give rise to a probabilistic metastable substate (PMS) space typifying each brain state [51–53]. A global brain state such as anesthesia and sleep is characterized by a given behavioral pattern and capacity for cognitive processing, while a metastable substate refers to different re-occurring patterns that arise and govern global brain states [54]. LEiDA has been shown to be robust and successful in identifying brain states in healthy aging [47, 55], depression [56] and different states of consciousness [5, 57, 58]. Interestingly, different clustering configurations have revealed overlaps between the BOLD phase-locking states (i.e., centroids) and fMRI resting-state networks [56, 58]. The model-based approach consists of building whole-brain models composed of a network of coupled local nodes to simulate the empirical PMS and artificially perturb the resulting PMS model to force the transition to a desired control state. This framework has been successfully applied in other brain states, showing transitions from aging [2], patients with depression [59] and schizophrenia [4] towards more healthy regimes.

Here, we aimed to study the dynamical complexity and causal mechanisms of brain activity in DoC by using the aforementioned framework. Firstly, we applied LEiDA to define the PMS of DoC patients and healthy controls. Secondly, we built Hopf whole-brain models fitted and optimized to the empirical PMS of DoC. This generative whole-brain model links structural anatomy with functional dynamics based on effective connectivity. Finally, we applied off-line *in silico* (i.e., artificial) unilateral and localized probing to force the transition from the PMS obtained in MCS and UWS, separately, to the PMS of healthy controls. We also evaluated the transition from UWS to MCS. In this way, employing off-line *in silico* probing, we could evaluate the effects of all potential local perturbations to the mechanistic global effects and sensitivity to stimulation.

## Materials and methods

### Ethics statement

The study was approved by the Ethics Committee of the Faculty of Medicine of the University of Liège according to the Helsinki Declaration on ethical research. Written informed consent was obtained from controls and the patients’ legal surrogates.

### Participants

A total of 23 controls and 45 non-sedated patients with DoC were selected from a dataset described in previous studies [13, 15, 16]. Trained clinicians carried out the clinical assessment and Coma Recovery Scale-Revised (CRS-R) scoring to determine the patient’s state of consciousness. The CRS-R diagnosis was made after at least 5 CRS-R, and the highest level of consciousness was taken as the final diagnosis, which was also confirmed using positron emission tomography (PET) (i.e., patients in MCS presented a relatively preserved metabolism in the frontoparietal network, whilst patients with UWS had a bilateral hypometabolism in this network). Thus, 29 patients in MCS and 16 in UWS were included. S1 Table can be consulted for patient demographics.

### MRI data acquisition

MRI data were acquired on a 3T Siemens TIM Trio scanner (Siemens Inc, Munich, Germany). Resting-state fMRI data were obtained using a gradient echo-planar imaging (EPI) sequence (300 volumes, 32 transversal slices, TR = 2000 ms, TE = 30 ms, flip angle = 78°, voxel size = 3x3x3 mm, FOV = 192 mm). After fMRI acquisition, a structural T1 magnetization-prepared rapid gradient-echo (MPRAGE) sequence was acquired (120 slices, TR = 2300 ms, voxel size = 1.0x1.0x1.2 mm, flip angle = 9°, FOV = 256 mm). Finally, diffusion-weighted MRI (DWI) was acquired with 64 directions (b-value = 1,000 s/mm^2^, voxel size = 1.8 × 1.8 × 3.3 mm^3^, FOV = 230 × 230 mm^2^, TR/TE = 5,700/87 ms, 45 transverse slices, 128 × 128 voxel matrix) preceded by a single b0 image.

### Resting-state fMRI pre-processing

The pre-processing of resting-state fMRI data was performed using MELODIC (Multivariate Exploratory Linear Optimized Decomposition into Independent Components) version 3.14 [60] from FMRIB’s Software Library (FSL, http://fsl.fmrib.ox.ac.uk/fsl) as described in our previous studies [15, 16]. The following steps were performed: discarding the first 5 volumes, motion correction motion using MCFLIRT [61], non-brain removal using BET (Brain Extraction Tool) [62], spatial smoothing with a 5 mm Gaussian Kernel, rigid-body registration, high pass filter (with a cutoff of 100 s) and single-session Independent Component Analysis (ICA) with automatic dimensionality estimation. Then, noise components and lesions-driven artifacts (for patients) were manually classified and removed for each subject by looking at the spatial map, time series, and power spectrum [63, 64] using FIX (FMRIB’s ICA-based X-noiseifier) [65]. Finally, FSL tools were used to co-register the images and extract the time series between 214 cortical and subcortical brain areas for each subject in MNI space from the Shen resting-state atlas (without the cerebellum) [66].

### Probabilistic tractography pre-processing

A whole-brain structural connectivity (SC) matrix was computed for each subject of the control group and then averaged in a two-step process as described in previous studies [67–69]. We used the resting-state atlas mentioned above to create a structural connectome in each individual’s diffusion native space. In brief, DICOM images were converted to Neuroimaging Informatics Technology Initiative (NIfTI) using dcm2nii (www.nitrc.org/projects/dcm2nii). The b0 image in DTI native space was co-registered to the T1 structural image by using FLIRT [70]. Then, the T1 structural image was co-registered to the standard space by using FLIRT and FNIRT [70, 71]. The transformations were inverted and applied to warp the resting-state atlas from MNI space to the native diffusion space by applying a nearest-neighbor interpolation method. Analysis of diffusion images was performed using the processing pipeline of the FMRIB’s Diffusion Toolbox (FDT) in FMRIB’s Software Library www.fmrib.ox.ac.uk/fsl. Non-brain tissues were extracted using Brain Extraction Tool (BET) [62], eddy current-induced distortions and head movements were corrected using eddy correct tool [72], and the gradient matrix was reoriented to correct for subject motion [73]. Then, Crossing Fibres were modeled using the default BEDPOSTX parameters, and the probability of multi-fibre orientations was computed to improve the sensitivity of non-dominant fibre populations [74, 75]. Probabilistic Tractography was performed in native diffusion space using the default parameters of PROBTRACKX [74, 75]. The connectivity probability to each of the other 214 brain areas was estimated for each brain area as the total proportion of sampled fibres in all voxels in the brain area *n* that reached any voxel in the brain area *p*. Given that Human Diffusion Tensor Imaging (DTI) does not capture directionality, the *SC_np_* matrix was symmetrized by computing its transpose *SC_np_* and averaging both matrices. Finally, to obtain the structural probability matrix, the value of each brain area was divided by its corresponding number of generated tracts.

### Leading Eigenvector Dynamics Analysis (LEiDA)

**Fig 1:**
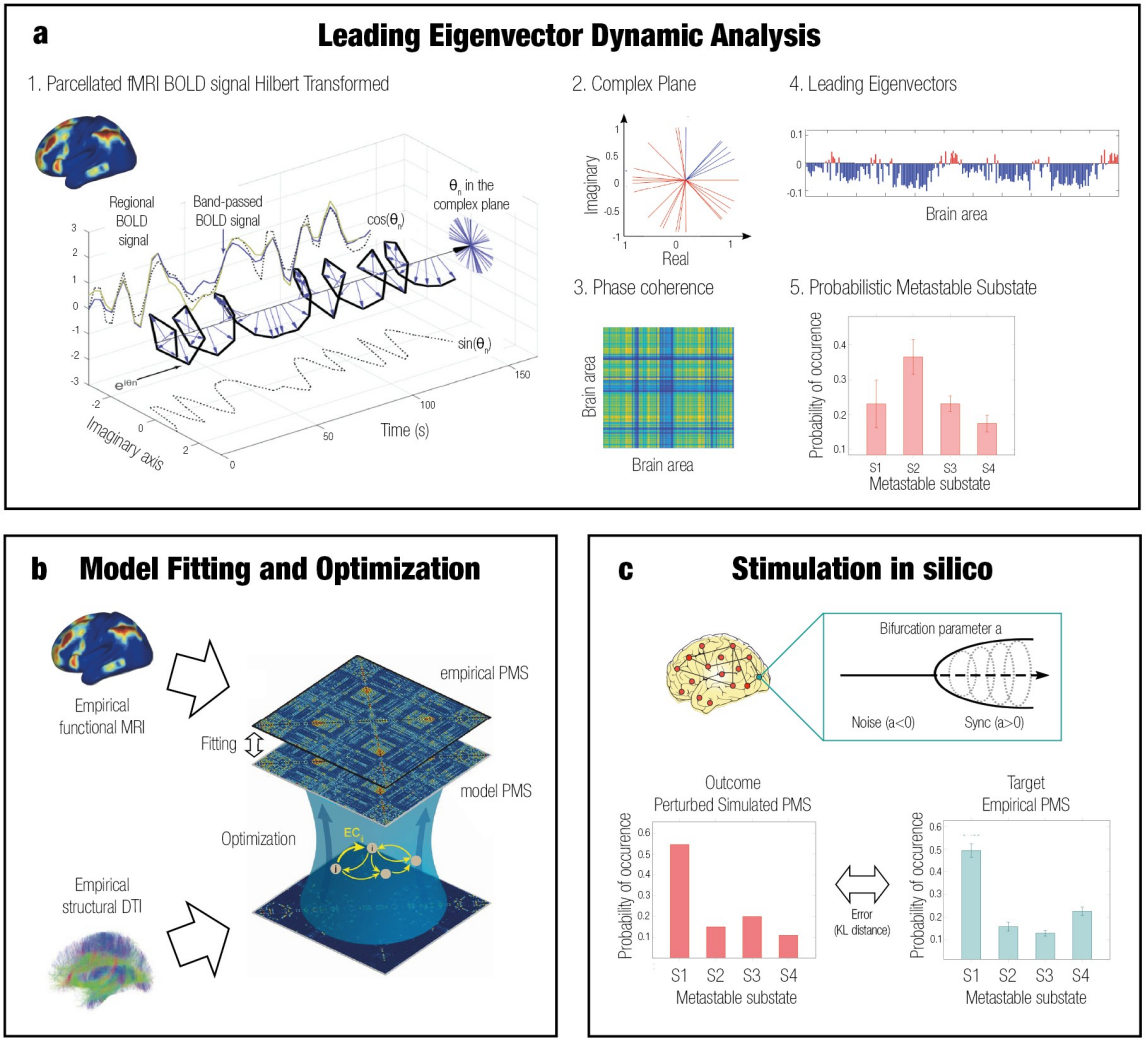
Overview of model-free and model-based frameworks. **a** Model-free framework: Leading Eigenvector Dynamic Analysis (LEiDA). The BOLD time signal for each of the 214 brain areas was band-passed filtered and Hilbert transformed. The complex plane shows the positive and negative real and imaginary components at a specific timepoint *t*. The phase coherence matrix dFC(*t*) between brain areas for each time window was calculated. Then, the leading eigenvector *V*_1_(t) capturing the principal orientation of the BOLD phase for each of the matrices was calculated for each time *t*—positive values in red, negative values in blue. The leading eigenvectors for all time points of all participants were clustered using K-means (*k* = 4), and the probability of occurrence of each of the cluster centers is shown in the Probabilistic Metastable Substate (PMS) Space. **b** Model-based framework: whole-brain model. A whole-brain model based on the frequency *w* of the empirical fMRI data and DTI was fitted to the empirical PMS space by calculating the value of the global coupling *G* that minimized the KL distance between the empirical and the simulated PMS. The model was optimized using the effective connectivity (EC) by adjusting each connection with a gradient descent approach until convergence. **c** Model-based framework: stimulation *in silico*. A transition was forced systematically from a source state to a target state by stimulating each brain area separately. The bifurcation parameter was shifted positively and negatively for synchronization and noise protocols, respectively. The optimal unilateral perturbation was obtained at the minimal KL distance between the stimulated modeled PMS and the target empirical PMS. Figure adapted from Deco et al., 2019 [5] under Creative Commons Attribution License 4.0 (CC BY).

This first step aims to define the empirical brain states from a quantitative point of view, defined as a conjunction of substates, applying LEiDA [47] as schematized in Fig 1A. For all subjects in all states, the blood oxygenation level-dependent (BOLD) time series of each brain area of the parcellation were filtered in the range 0.04–0.07 Hz and Hilbert-transformed to obtain the evolution of phase of the time series. A BOLD phase coherence matrix dFC(*t*) was then calculated at any given repetition time (TR) between each brain area pair *n* and *p* by calculating the cosine of the phase difference as:

$$\begin{array}{c} dFC(n,p,t)=\text{cos}\;\left(\theta (n,t)-\theta (p,t)\right). \end{array}$$ (1)

In this way, the interregional BOLD signal synchrony for all subjects was obtained at all time points. If nodes are temporarily aligned, the difference between their Hilbert transformed signal angle is 0°and the phase coherence is close to one [cos(0°) = 1]. When a pair of nodes show orthogonal BOLD signals, the phase coherence is close to zero [cos(90°) = 0]. The resulting dFC(*t*) of each subject was a 3D matrix of size of N × N × T, being N the number of brain areas (214) and T the total time points (295). A total of 68 3D matrices were calculated, corresponding to all of the groups together (controls, MCS and UWS).

In order to facilitate the future classification process, the dominant connectivity pattern was obtained by reducing the dimensionality of the matrices into their leading eigenvectors *V*_1_(t). This can be applied since FC matrices are undirected and symmetric across the diagonal [5]. The leading eigenvectors (of dimension N × 1) capture the dominant connectivity pattern at each time point *t* whilst explaining most of the variance, representing the contribution of each brain area to the whole structure and improving the signal-to-noise ratio [47]. The dimensionality of the data was reduced from N × N to N × 1, and the dominant functional connectivity pattern dFC(*t*) could be observed by calculating the outer product of V_1_(t) with its transpose (V_1_.V_1_.V^T^) [76].

The following step consisted of identifying recurrent FC patterns representing the substates. The leading eigenvectors dFC(*t*) for each TR and all subjects from all states (20060 = 68 participants * 295 timepoints) were clustered with K-means clustering, varying *k* from 3 to 8. This algorithm is an unsupervised method consisting of assigning the data to the closest cluster centroid iteratively and re-calculating the *k* centroids in each iteration until convergence. The resulting cloud centroids V_c_(t) represent the dominant connectivity pattern in each cluster. The *k* discrete number of patterns of size N × 1 correspond to the substates obtained from all subjects in all collapsed groups of subjects. These cluster centroids V_c_(t) represent the contribution of each brain area to the community structure and were rendered onto brain maps using the Surf Ice software (https://www.nitrc.org/projects/surfice/) and Human Connectome Workbench (https://www.humanconnectome.org/software/connectome-workbench).

Upon computing the discrete number of FC patterns for each *k*, we calculated the resulting probability of occurrence in each group. This was computed as the ratio between the total number of epochs assigned to a specific cluster (i.e., for each subject in each group divided by the total amount of epochs in the given group). This gave rise to the Probabilistic Metastable Substate Space (PMS), which typifies each brain state from the probability of occurrence of being in each particular substate from the substate repertoire.

### Whole-brain computational model

After characterizing the empirical PMS for the different profiles, a whole-brain Hopf computational model was obtained for each DoC state at the group level (Fig 1B). The dynamics from functional interactions between each brain area were emulated based on the anatomical SC. In other words, the emergence of activity can be explained in a mechanistic way by merging anatomical connectivity, which determines structure, and functional connectivity that represents activity dynamics, with the inclusion of effective connectivity (EC) [6]. The working point of each model was fitted to the empirical data and optimized by determining the specific parameters of the model [5].

The normal form of supercritical Hopf bifurcation (Landau-Stuart oscillator) was used to simulate the BOLD activity for each of the 214 cortical and subcortical brain areas based in Shen parcellation. The Landau-Stuart oscillator has been used to study transitions from noisy to oscillatory regimes and, when coupled based on the brain’s architecture, to replicate complex interactions in brain dynamics.

An uncoupled node *n* can be represented in Cartesian coordinates with the following pair of coupled equations:

$$\begin{array}{c} \begin{array}{c} \frac{\text{d}x_{n}}{\text{d}t}=\left[a_{n}-x_{n}^{2}-y_{n}^{2}\right]x_{n}-\omega_{n}y_{n}+\beta \eta_{n}\left(t\right),\\ \frac{\text{d}y_{n}}{\text{d}t}=\left[a_{n}-x_{n}^{2}-y_{n}^{2}\right]y_{n}+\omega_{n}x_{n}+\beta \eta_{n}\left(t\right), \end{array} \end{array}$$ (2)

where *x_n_* emulates the BOLD signal of the node and *η*_*n*_(*t*) is the additive Gaussian noise with standard deviation *β* = 0.01. The frequency of the system *f*_*n*_ = *ω*_*n*_/2*π* was estimated from the empirical data as the averaged peak frequency of the filtered BOLD signal in the 0.04- to 0.07-Hz band for each brain node *n* = 1, …, 214 [5]. Furthermore, the local bifurcation parameter *a* determines the solutions of the system governing the dynamics, in which a supercritical bifurcation is found at *a* = 0. For *a<0*, the node is stable in a fixed point and represented by low activity noise from the asynchronous firing of neurons. For *a>0*, metastable oscillations are obtained due to the synchronized firing of neurons at a frequency of *w/2π* [77]. Here, we chose a value of *a_n_* = -0.02 for each brain node *n* following previous findings [22]. Fluctuating stochastically structured signals that preserve resting-state network structure were found in this subcritical regime.

The whole-brain dynamics were modeled by including an additive coupling term *C_np_* which adjusts the input to node *n* from each of the rest of the nodes *p* based on the SC. This weighted matrix assumes different myelination densities across long-rage connectivities. A global coupling weight *G* was also added to represent the strength between all nodes, corresponding to the control parameter adjusted to fit the dynamical working region of the simulations to the empirical data. It scales all of the connections allowing maximal fitting between simulations and empirical data. The whole-brain dynamics at each node *n* was thus defined by the following set of coupled equations [51]:

$$\begin{array}{c} \begin{array}{c} \frac{\text{d}x_{n}}{\text{d}t}=\left[a_{n}-x_{n}^{2}-y_{n}^{2}\right]x_{n}-\omega_{n}y_{n}+G\sum_{p_{=1}}^{N}C_{np}\left(x_{p}-x_{n}\right)+\beta \eta_{n}\left(t\right),\\ \frac{\text{d}y_{n}}{\text{d}t}=\left[a_{n}-x_{n}^{2}-y_{n}^{2}\right]y_{n}+\omega_{n}x_{n}+G\sum_{p_{=1}}^{N}C_{np}\left(y_{p}-y_{n}\right)+\beta \eta_{n}\left(t\right). \end{array} \end{array}$$ (3)

#### Model fitting: Comparing empirical and simulated probability metastable space states

For optimal spatiotemporal fit of whole-brain models to their empirical PMS space, the value of *G* was ranged from 0 to 0.5 in steps of 0.01, and the model was iterated 250 times. LEiDA was computed to the Hilbert-transformed simulated signal using the centroids already defined by the empirical substates in order to compute the simulated PMS space. Each model was fitted to the empirical data by deciding which value of *G* approximated it better [5]. This corresponded to the lowest Kullback-Leibler (KL) distance between the empirical and simulated probabilities of each substate [5], given by:

$$\begin{array}{c} KL(P_{emp},P_{sim})=0.5(\sum_{i}\;P_{emp}(i)\;\text{ln}\;(\frac{P_{emp}(i)}{P_{sim}(i)})+\sum_{i}\;P_{sim}(i)\;\text{ln}\;(\frac{P_{sim}(i)}{P_{emp}(i)})), \end{array}$$ (4)

where *P_emp_(i)* and *P_sim_(i)* are the empirical and simulated probabilities, respectively, of metastable substate *i*.

#### Model optimization: Method for updating effective connectivity

After defining the value of *G* of each model, the models were optimized separately and the SC was updated in order to access potential missing connections. The initial value of *C* for each of the models was provided by a primer empirical DTI structural connectivity corresponding to the average of control subjects [6]. Specifically, *C* was initially normalized to a maximum value of 0.2 in order to have the same range of values as in previous works [5, 51]. The SC was then transformed to effective connectivity (EC) in an iterative manner by calculating the distance between the grand average phase coherence matrices of the model *FC_ij_^phases_mod^* and the empirical matrices *FC_ij_^phases_emp^*. Each structural connection between different nodes *i* and *j* was adjusted with a gradient descent approach given by:

$$\begin{array}{c} C_{ij}=C_{ij}+\epsilon (FC_{ij}^{phases\_emp}-FC_{ij}^{phases\_mod}), \end{array}$$ (5)

where *ϵ* = 0.01, and the grand average phase coherence matrices are defined:

$$\begin{array}{c} FC_{ij}=\langle \text{cos}(\varphi_{j}\left(t\right)-\varphi_{i}\left(t\right))\rangle , \end{array}$$ (6)

where *φ*(*t*) denotes the Hilbert transform BOLD signal phase of the nodes *j* and *i* at time *t*, and the brackets indicate the average across time. This was repeated until the difference between the empirical and simulated values was smaller than 0.001 [5].

### Unilateral perturbation of the whole-brain model

After building the models, the transitions from each DoC state to control and from UWS to MCS were studied (Fig 1C). The models for DoCs were stimulated *in silico* by moving locally in a unilateral way the local bifurcation parameter *a* of each of the 214 brain areas, while maintaining the rest at their initial bifurcation value (i.e., *a* = -0.02). Different intensity levels were applied area by area under the protocols of synchronization and noise. The protocols were represented by the sign of the local bifurcation parameter, and the stimulation intensities by the absolute value of each step [51]. The bifurcation parameter *a* of each brain area was shifted positively in the synchronization protocol, representing a stable limit cycle oscillation, from -0.02 to 0.18 in steps of 0.01. For the noise modality, it was shifted negatively, representing a low activity noisy state [22], from -0.02 to -0.22 in steps of -0.02. Each simulation was repeated 10 times and the results were averaged to minimize random effects from the Gaussian noise of the model [5]. The global brain dynamics of each local and independent stimulation were evaluated by calculating the PMS based on the empirical cluster centroids initially obtained. The fitting to the target states was measured by calculating the KL distance (described in the previous section) between the probabilities of each substate of the simulated DoC models separately, which are the source, and the empirical target PMS. The areas more prone to promote a desired transition after simulation were detected from the ones presenting the lowest KL distance.

#### Statistical analysis

Statistical analysis was performed using MATLAB R2022a software from MathWorks (Natick, MA, USA). Permutation-based Wilcoxon tests with 1000 iterations were used to test the results of the LEiDA method, specifically the probability of occurrence of the whole range of explored clustering conditions (*k* from 3 to 8). The Wilcoxon test was used to compare each permutation with a significance threshold of 0.05. We applied the False Discovery Rate (FDR) method [78] to correct for multiple comparisons when testing the differences between groups (controls, MCS, and UWS) and the number of cluster centers (i.e., substates). All p-values shown correspond to the differences that remain significant after FDR correction.

## Results

### LEiDA

**Fig 2:**
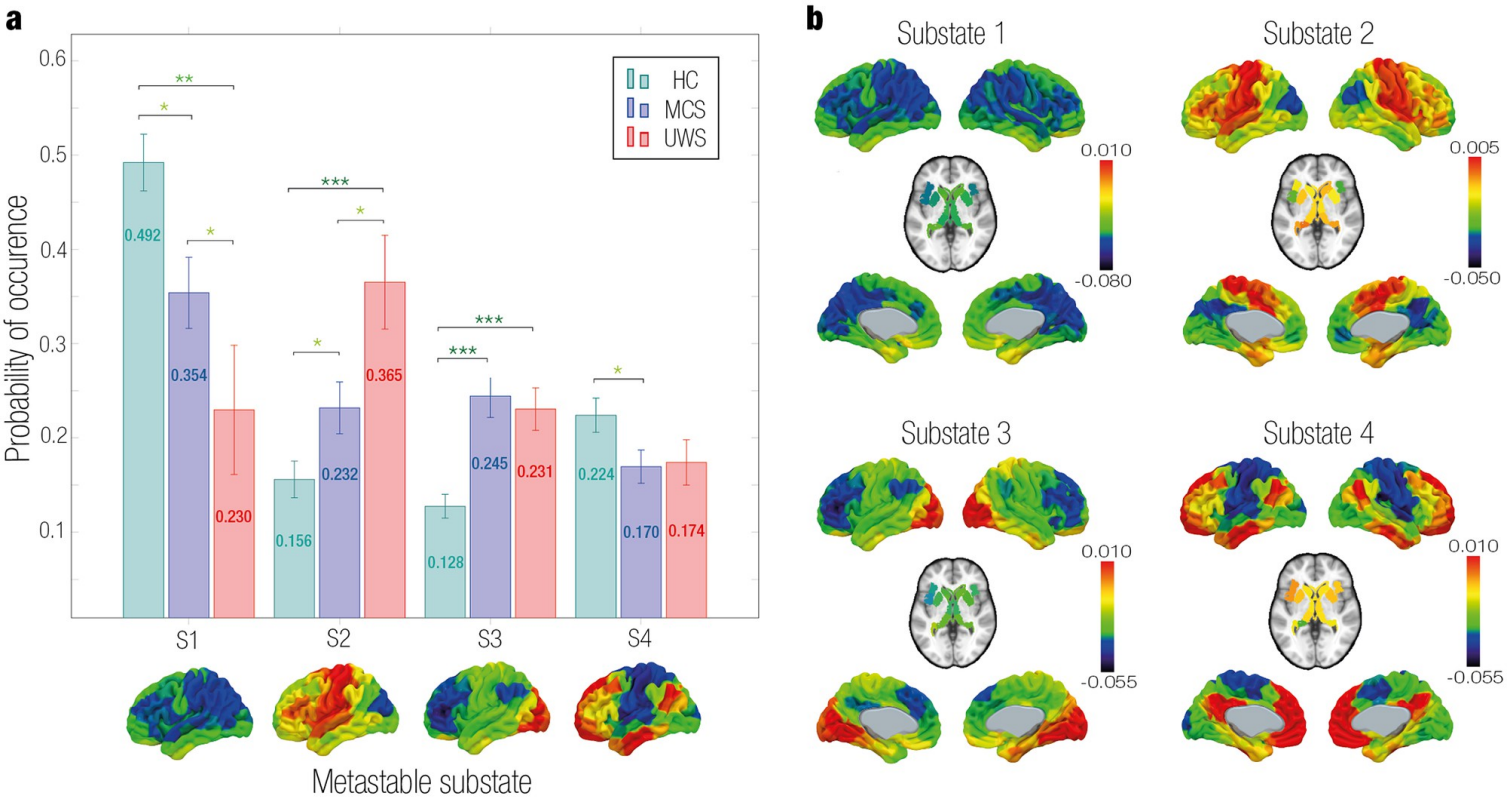
Model-free results: Empirical Probabilistic Metastable Substate (PMS) Space. **a** Probability of Occurrence. The mean probability of occurrence for each group in each substate was calculated with a 95% confidence interval. The substates 1 and 4 had a higher probability of occurrence for the control group compared to DoC. Substates 2 and 4 had a lower probability of occurrence for the control group compared to DoC. Statistically significant differences are represented with asterisks (* p < 0.05, ** p < 0.01 and *** p < 0.001). **b** Rendered brains represent the leading eigenvectors of each substate. Substate 1 was characterized by all elements of the eigenvector with the same sign. Substate 2 had a functional community formed by areas in the somatomotor network. Substate 3 presented a local coordination in the occipital lobe (visual network). Substate 4 showed coordination in brain areas from the medial-frontal network, fronto-parietal network and DMN.

We selected the minimum number of clusters (*k*) that statistically differed between the three groups. Empirical results for all clustering configurations can be found in S1 Fig. The configuration that best described the empirical data across all participants and distinguished between groups was detected at *k* = 4. The probability of occurrence for the PMS of each group is visualized in Fig 2A and the cluster centroid eigenvectors are rendered onto brain maps in Fig 2B. The leading eigenvectors had positive and negative signs partitioning the network into communities as red and blue colors, respectively. The strength of the color describes the strength with which each area belonged to the placed community [47].

The first substate presented the same sign for all eigenvector elements. The probability of occurrence was higher in controls [0.492 ± 0.030 (mean ± standard error)] compared to MCS [0.354 ± 0.038, *P* = 0.016] and UWS [0.230 ± 0.069, *P* = 0.004]. Furthermore, the probability was lower in UWS than in MCS [*P* = 0.034]. The rest of the substates (i.e., substates 2, 3, and 4) were characterized by subsets of brain areas that disengaged from the whole-brain network aligning with each other. In substate 2, central areas (somatomotor network) represented a pattern of activation. In this substate controls had the lowest probability of occurrence [0.156 ± 0.020] compared to MCS [0.232 ± 0.027, *P* = 0.037] and UWS [0.365 ± 0.050, *P*<0.001]. Moreover, the probability was higher in UWS than in MCS [*P* = 0.021]. Substate 3 exhibited a functional network led by the occipital lobe (visual network). In controls, the probability of substate 3 was lowest [0.128 ± 0.013] compared to MCS [0.245 ± 0.023, *P*<0.001] and UWS [0.231 ± 0.023, *P*<0.001]. This substate did not discriminate significantly between DoC groups. Substate 4 had a coordination between areas mainly of the medial-frontal network, fronto-parietal network and DMN. This metastable substate only discriminated between controls [0.224 ± 0.018] and MCS [0.170 ± 0.018, *P* = 0.028].

As a further analysis, we calculated the Kuramoto order parameter of each brain state, a measure that captures the degree of synchrony of a system [79]. Results showed statistically significant differences in all comparisons, revealing the highest values (i.e., more synchronization) for controls compared to MCS and UWS in decreasing order (S2 Fig). In addition, we looked for the correlation between the CRS-R of DoC and the probability of occurrence of each substate without finding any significant relationship (S3, S4 and S5 Figs).

### Fit whole-brain computational model to the brain states of DoC groups

**Fig 3:**
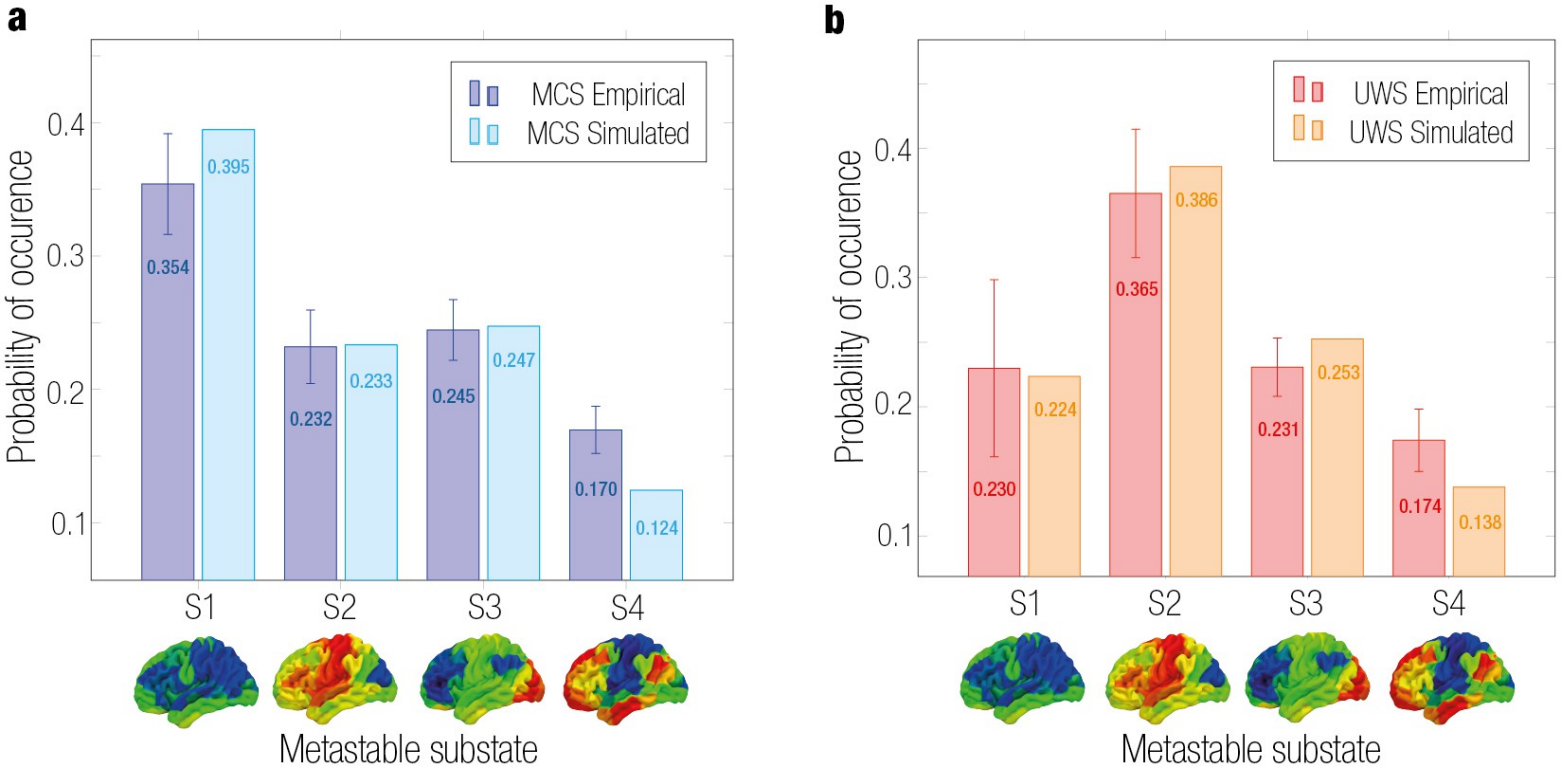
Model-based results: Whole-brain model fitting and optimization. Comparison between empirical and simulated PMS of each group. Optimal fit was given by the minimal KL distance value corresponding to a global coupling weight of **a** *G* = 0.07 for MCS and **b** *G* = 0.04 for UWS.

For the MCS and UWS groups, we fitted the PMS to a causal mechanistic whole-brain model. We optimized and adjusted the models in order to select the parameters that displayed the most approximate regime to empirical PMS (see Materials and methods). The best fit between the empirical and simulated PMS was found at *G* = 0.07 and *G* = 0.04 for MCS and UWS models, respectively (Fig 3). Evolution of the KL distance as a function of G and the EC of the optimal fit for both models can be found in S6 Fig.

### *In silico* stimulations to force transitions

Following model fitting and optimization, we systematically perturbed the PMS model of each DoC group and compared it with the empirical PMS of the control group. We also evaluated the transition from UWS to MCS. Each brain node was shifted by increasing the absolute value of the bifurcation parameter *a* while maintaining the rest at *a* = -0.02. The absolute value of the bifurcation parameter represents the intensity of stimulation. A synchronization protocol was addressed with positive values, and a noise protocol with negative values. In each stimulation, the global brain dynamics of the perturbed model were evaluated by calculating the PMS using the cluster centers obtained from the empirical LEiDA. Transitions were evaluated with the KL distance, which measures the difference between two probability distributions (i.e., the PMS of the perturbed source state and the PMS of the empirical target state). Optimal transitions were identified as the brain areas with the minimum KL values across the whole brain. In other words, the KL distance with the lowest value represents the optimal transition, while higher KL values represent suboptimal perturbations.

**Fig 4:**
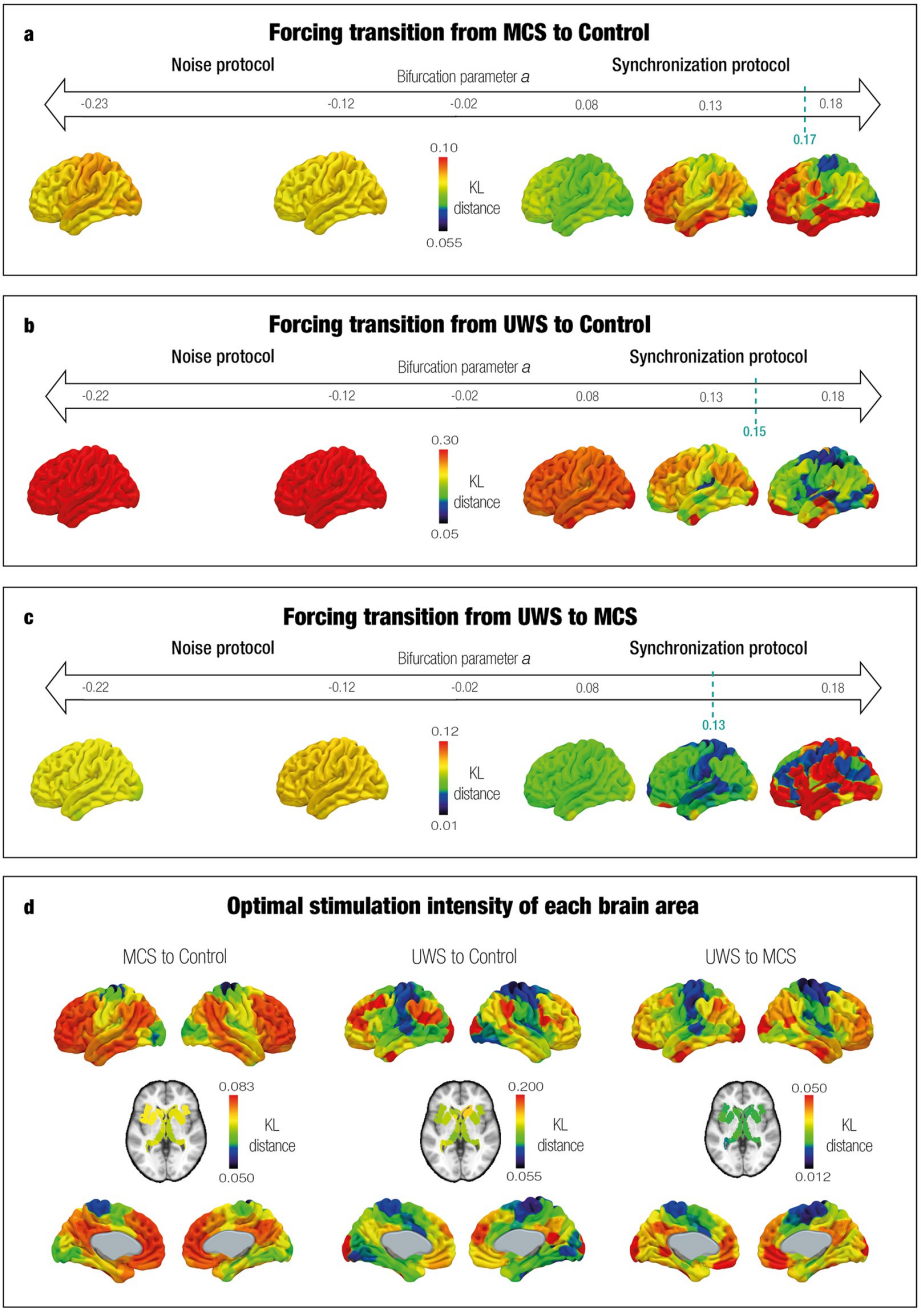
Model-based results: *In silico* probing to force transition from DoC to control target state. We used synchronization and noise stimulation protocols to shift the local bifurcation parameter. The strength of the unilateral perturbation corresponds to the absolute value of the bifurcation parameter and the sign to the modality (synchronous with positive values, noise with negative values). The x-axis shows the stimulation intensity (from softer to stronger), and the color scale represents the KL distance. The best effectiveness was found where KL distance was minimal. For both DoC (**a** and **b**), the synchronization protocol forced a transition to the control state. The same occurred for UWS towards MCS (**c**). This can be observed with the lower KL distance when increasing values of the local bifurcation parameter in a positive manner. The left sides of the x-axis show that the noise protocol presented poor effectiveness given that KL distances were higher than in the synchronization protocol. **d** Shows the KL distance rendered onto brain maps with the optimal stimulation for each brain area in the synchronous protocols. The color scale represents the KL distance given by the best stimulation, with the lowest values corresponding to the somatomotor and some subcortical areas (the best targets).

The results of the *in silico* stimulation with different protocols and intensities for MCS and UWS are shown in Fig 4A, 4B and 4C. The color scale represents the KL distance between the perturbed PMS and the target PMS after stimulating each individual brain area separately. The best fit is indicated by a lower KL distance, note that the color scales are different for each DoC condition, adjusted accordingly for better resolution. For the synchronization protocol, a successful transition was forced from the source states of MCS and UWS to the control state. We can observe that for MCS most regions promoted a transition with lower stimulation intensity compared to UWS. We computed the correlation between the optimal transition of each brain area to the control state in MCS and UWS. Results reflected their regional patterns were spatially correlated (S7 Fig). In addition, the transition from UWS to the empirical MCS state was also achieved in the synchronization protocol. This followed the same trajectory as in the perturbation of UWS to control, with lower stimulation intensities for the optimal fit. In contrast, in the noise protocol, the KL distance did not decrease for both MCS and UWS (i.e., colors are red and yellow rather than green and blue). This means that as a result of applying a noise protocol, the transitions from DoC to a control target state and from UWS to MCS were not possible, evidenced by poorer fit.

In the synchronization protocol, a transition was likely to occur in many areas if sufficiently stimulated. Fig 4D illustrates the rendering of the KL distance between the perturbed PMS and the target PMS after stimulating each individual brain area separately, at their particular optimal stimulation intensity. Areas in the somatomotor network were the most sensitive ones, as well as the insula and precuneus, provoking transitions in both cases (MCS and UWS) (S2, S3 and S4 Tables). Concerning the transition from DoC to a control state, the thalamus was also relevant. For MCS, more sensitivity was found in a right segment with highest probability of connection with the posterior parietal cortex, and secondly a right segment with highest probability of connection with the prefrontal cortex. For both DoC states (i.e., MCS and UWS) a particular sensitive segment was found in the left thalamus, with the highest probability of connection with the prefrontal cortex, premotor and posterior parietal cortex. S5 and S6 Tables can be consulted for thalamic node labels and their overlap with Oxford Thalamic Connectivity Atlas [75] and AAL structural parcellation [80], respectively. S8 Fig shows the rendered thalamic node segments. As a further analysis, we found that brain areas with higher sensitivity to perturbation showed stronger similarities to the anatomical connections of healthy controls. By contrast, those regions showing lower similarities to the anatomical connections of healthy controls were not optimal for perturbation (S9 Fig). This reveals that better-preserved brain areas in DoC are more suitable for reversing the dynamics of the system. The relation is not direct, given that the arrangement of better-preserved brain areas does not exactly align with the most sensitive ones.

**Fig 5:**
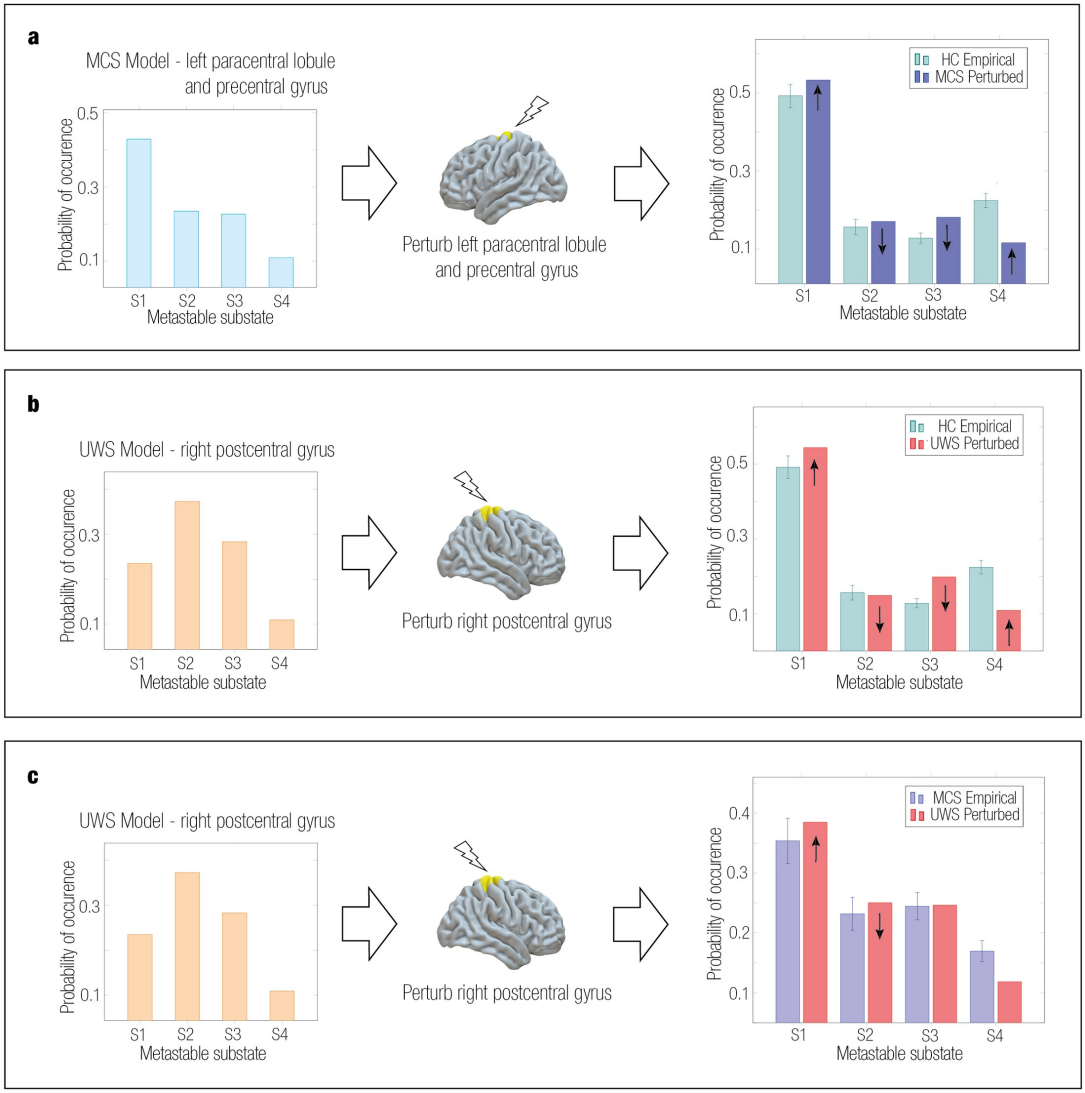
Comparison between perturbed PMS of MCS and UWS groups. We show the simulated and perturbed PMS for DoC groups and the empirical target PMS. For all transitions, the synchronization protocol increased the probability of the first and last substates and decreased the probability of the other substates, consistent with the empirical PMS of the control group. **a** Simulated MCS had a best approximation to the PMS of controls by perturbing unilaterally the left paracentral lobule and precentral gyrus at *a* = 0.17. **b** Simulated UWS had the best approximation to the PMS of controls by perturbing unilaterally the postcentral gyrus (right) at *a* = 0.15 **c** Simulated UWS had the best approximation to the PMS of MCS by perturbing unilaterally the postcentral gyrus (right) at *a* = 0.13.

Specifically, the best fit to the control PMS space was obtained when stimulating the left paracentral lobule and precentral gyrus, with a bifurcation parameter value of 0.17 for MCS and the right postcentral gyrus with a bifurcation parameter value of 0.15 for UWS. Furthermore, the best fit from the UWS to MCS was achieved when stimulating the right postcentral gyrus with a bifurcation parameter value of 0.13. As a result, the perturbed and target probabilities were very similar in all four metastable substates of the PMS Fig 5. Statistically significant results in terms of the perturbation can be consulted in S10 Fig. For the optimal transitions, the evolution of the PMS as a function of the stimulation intensity was gradual until the optimal perturbation was achieved (S11 Fig).

## Discussion

We successfully applied model-free and model-based approaches to find causal evidence for the brain dynamics in DoC and transitions to a control state applying the awakening framework [5]. Firstly, we significantly distinguished between brain states by characterizing the PMS of DoC and controls using LEiDA. For each group, we identified metastable substates (i.e., dynamic patterns) with an associated probability of occurrences and alternation profiles [47]. We then fitted a Hopf model to each empirical PMS for each DoC state. In this way, we were able to force transitions in DoC towards a control target state using exhaustive *in silico* perturbations. Finally, by varying stimulation intensities, we revealed how changes in local brain areas using a synchronous modality can reshape whole-brain dynamics in DoC. In this way, we could determine the mechanistic global effects of local perturbations and the most sensitive areas in terms of their perturbability.

In the model-free approach, using LEiDA, we identified substates with network-specific changes whose probabilities varied in each brain state (Fig 2). In particular, we found controls were more able to access substates 1 and 4. Substate 1, in which all BOLD signals followed the leading eigenvector, has been shown to exist in previous LEiDA studies [56, 58, 81]. This substate has been associated with a global state [82], synchronized stability [83], or noise artifacts [84]. Furthermore, we found substate 4 had a coordination of areas overlapping the medial-frontal network, fronto-parietal network and DMN. The medial-frontal network is related to cognitive processing, emotional regulation, and social cognition [85, 86]. A higher probability of occurrence of this substate in controls could be related to increased cognitive and social processing, which are characteristics associated with a healthy brain state. In addition, the fronto-parietal network has been found essential for cognitive control and goal-driven behavior coordination [87]. Altered connectivity in this network has been detected in DoC [88, 89]. Lastly, the DMN, responsible for internal self-related and external perceptual awareness, autobiographical memory and mind-wandering, has been shown disrupted in patients with DoC [29, 90–94]. In line with the decreased probability of occurrence of this substate in DoC, deactivation of the DMN has been identified as a marker of consciousness, reflecting disrupted introspection [95].

On the other hand, our results show DoC patients were more likely to be in substates 2 and 3. Firstly, substate 2 exhibited a functional network led by the somatomotor network, involved in processing bodily sensations, and motor planning and execution [96]. In line with our results, higher functional network centrality has been identified in the sensorimotor network in DoC, highlighting this network becomes more relevant in patients possibly due to communication impairments [97]. Furthermore, higher functional connectivity has been revealed in the sensorimotor network during light sedation [98]. In contrast, other investigations identified lower connectivity in somatomotor and sensory networks in DoC [99, 100]. Lastly, substate 3 was led by the visual network. Interestingly, a study found a correlation between CRS-R visual score and the visual network integrity [101].

In the model-based approach, we modeled brain activity as a system of non-linear Stuart-Landau oscillators (also known as Hopf bifurcation) to link the underlying anatomy with functional dynamics [51]. Hopf models have allowed simulating several brain states in health and disease with high fitting accuracy [5, 54, 102]. These models have been able to capture both local and global brain dynamics, while having lower computational costs than more detailed models such as spiking neurons [103, 104]. When fitting and optimizing the models to the empirical data, the dynamics of each brain area were set in the subcritical regime (*a_n_* = -0.02). This represented fluctuating stochastically structured signals allowing for fluctuations between noise and oscillation, while still maintaining resting-state network structure [22]. We observed that the MCS group had a higher value of global coupling weight *G* than the UWS (Fig 3). This parameter represents the relationship between local and global brain dynamics and the effects of structural connectivity on brain dynamics. The greater the value of *G*, the less restricted the brain network interaction is to areas with high structural connections. This goes in line with previous studies, where MCS showed more propagation of brain activity and connectivity between distant brain areas than UWS [15, 16].

By combining the model-based approach with *in silico* stimulations, we explored artificial brain transitions between different states. This strategy allowed us to find the optimal areas to stimulate and re-balance the underlying brain dynamics in patients with DoC towards more healthy states. Thus, *in silico* stimulation enabled testing exhaustive trials without the ethical constraints of real-world experiments [40, 51]. We shifted the brain dynamics’ landscape rather than the working point, ensuring propagation, facilitating plasticity, and targeting a system reorganization [5, 30]. The bifurcation parameter was independently shifted in each brain area, affecting the whole-brain dynamics. We applied synchronization and noise protocols with positive and negative intensities of the bifurcation parameter, respectively. The synchronization protocol shifted the system towards self-sustained oscillations, and the noise protocol towards a stable point with low noisy activity [39]. We evidenced transitions from DoC states to more healthy regimes using the synchronization protocol, since the KL distance between the perturbed PMS and the target PMS decreased in this modality (Fig 4). By contrast, the noise protocol led to higher KL distances, i.e., poor fit, indicating that the transition was not possible. We then estimated the Kuramoto order parameter for each state, revealing higher levels of synchrony in controls compared to DoC [79], supporting our perturbational results. The healthy brain operates near criticality, a state between chaoticity and synchrony, characterized by flexibility and efficient information processing [105, 106]. Furthermore, abnormalities in DoC reduce the ability to integrate and synchronize areas to process information [107, 108]. Thus, considering synchronous oscillations have a role in neuronal communication and long-range functional connectivity between brain areas [109, 110], increasing the synchrony level might help re-balance the dynamics in DoC. A further finding was that for the transition from DoC to the control state, MCS exhibited higher sensitivity to external perturbations than UWS. Metastable substates with the highest probability of PMS spaces in both DoC groups shifted from substates with subsets of brain areas aligned within each other in the somatomotor network (substate 2) and visual network (substate 3) to a substate dominated by regions from the medial-frontal network, fronto-parietal network and DMN (substate 4), and to a substate with global brain activity (substate 1) (Fig 5). Lastly, in line with previous findings [54], the transition from UWS to MCS revealed the same trajectory towards the control state and a similar sensitivity pattern across brain areas.

In terms of brain areas promoting a transition, most were found in the somatomotor network, overlapping substate 2 from LEiDA results. Consequently, perturbing its leading functional areas would reduce the probability of this substate (i.e., approximate to a control state). Particularly, the most sensitive area for MCS corresponded to the left paracentral lobule and precentral gyrus. These belong to the primary motor area in which the former subserves motor functioning [111] and the latter is associated with motor speech production [112, 113]. Interestingly, repetitive TMS in the left primary motor cortex in a patient with MCS showed improved arousal and awareness [114]. Contrarily, a patient with chronic UWS did not show positive outcomes [115]. Furthermore, we found that for the transition from UWS to MCS and control, the postcentral gyrus was the best target. This region has been associated with impaired somatosensory functions [100] and found to distinguish DoC patients by its weighted global connectivity [116]. A study found a correlation between favorable outcomes in DoC with structural connectivity between the thalamus, putamen and somatomotor network (in the left hemisphere) [117]. This subnetwork was associated with voluntary movement and underlying consciousness, and our results support the hypothesis that this subnetwork plays an important role in the recovery process. In addition, the study also identified the postcentral gyrus as the node with the highest number of edges within the subnetwork. This may support our observations, suggesting that disturbances in the postcentral gyrus could have a greater influence on overall brain dynamics compared to other areas. Lastly, other sensitive areas were the precuneus and insula. A study identified the anterior precuneus as a hub in recoverable unconscious states (i.e., sleep, anesthesia), including patients who emerged from UWS [118]. The same investigation showed a correlation between its degree of centrality with Glasgow Outcome Scale scores, associating this area as a sensorimotor center for preserving functional integrity crucial for recovery. On the other hand, the anterior insula has been hypothesized as having a role of awareness maintenance, and recovery was related to increased connectivity of the dorsal agranular insula with the bilateral inferior parietal lobe and temporal pole [119].

Finally, the thalamus was relevant in the transition of DoC, specifically to the control state. This holds significance in DoC studies given the widespread interest in thalamic stimulation as a treatment [120–122]. The thalamus has been associated with primary brain functions, and identified to play an integral role in the widely adopted mesocircuit model. This model postulates feedforwarding connections between the thalamus and cortical regions for supporting conscious processing and identifies the thalamus as a hub for receiving sensory information [123]. In this context, increased inhibition and decreased excitatory output of the thalamus have been identified as a hallmark in DoC [124, 125], with the highest relevance in the thalamo-frontal connection [126, 127]. Interestingly, a study revealed an increased degree of centrality of the thalamus in DoC patients compared to controls associating it to compensatory mechanisms and a reorganization of network structure [128]. This higher coupling and communication of the thalamus with other brain areas might explain the broadcasting of the local stimulation in the thalamus at the global level dynamics, as revealed in our results. Furthermore, published evidence indicates that the thalamus possesses distinctive nuclei characterized by different projection patterns, target cortical regions, and specialized functions [129–131]. This construct has been supported empirically, in which DBS in intralaminar nuclei reversed general anesthesia [132–134], anterior thalamus showed improvements in epilepsy patients [135, 136]s and central thalamus regulated arousal levels in healthy non-human primates [137]. In the case of DoC recovery, behavioral improvement in patients was observed after thalamic stimulation [120], particularly in the intralaminar thalamic nuclei [121] and central lateral nucleus thalamus [122]. Concerning our results, sensitive segments of the thalamus were those projecting to the prefrontal, premotor, and posterior parietal cortex. The prefrontal thalamo-cortical connectivity is crucial for the aforementioned mesocircuit model, whereas the premotor thalamo-cortical connectivity could be associated with the recovery of the patient’s limited and fluctuating awareness. An investigation found connectivity differences in frontal, premotor, sensorimotor and temporal thalamocortical connectivity among DoC [138]. Lastly, in the case of the posterior parietal thalamo-cortical connectivity, past research related bidirectional interaction between the thalamus and the posterior cortex (including the parietal cortex), to differences in consciousness levels in awake, sleeping, and anesthetized macaques [139].

Collectively, our efforts try to move forward in the study of empirical brain differences and model-based biomarkers in DoC. We want to underscore that our ultimate goal was not mainly to force an artificial transition from DoC to a control state per se but to elucidate the implications of external perturbation for a sensitivity assessment. This way, we evaluated the sensitivity in terms of the perturbability to *in silico* stimulation, ensuring causal mechanistic underpinnings of whole-brain reactivity to local perturbations. As a result, we examined each brain area independently, thus interpreting the perturbations in relative terms rather than absolute values. In addition, the main aim of computational neuroscience is to understand the causal mechanisms of a given brain state with reverse engineering [45]. This approach allows studying underlying dynamics through analytical tools rather than relying on statistical predictions. Consequently, the concept of overfitting is not applicable in this context. The focus lies on tuning the parameters of models in order to approximate empirical BOLD data as closely as possible. This allows the evaluation of underlying aspects which gives rise to the observed data while constraining the level of complexity of the model [140]. Such is the case in our Hopf brain models of the global coupling weight *G* representing the global scaling [51], and the additive coupling term *C* corresponding to the input received in each node from every other node [30]. The models are designed to maximally reflect empirical brain dynamics while summarizing features [45]. Therefore, our results are exploratory, still indicating a promising direction in DoC research. The breach in clinical settings could be reduced by moving forward with higher-quality data and high-standard theoretical contexts, confirming or contradicting theoretical transitions with empirical ones. This way, it can help to advance the translation from theoretical neuroscience to the clinic [108]. In the long term, this could motivate external perturbation as a therapeutic intervention for re-balancing the dynamics of post-coma patients towards healthier regimes.

Our work presents several limitations. As in previous works, we used the structural connectivity matrix estimated from healthy participants to build the DoC models [16, 54]. Considering that DoC patients presented heterogeneous lesions, the average from healthy participants was a reasonable starting point. Furthermore, the effective connectivity optimized the links between brain areas in each model, thus improving the relevant effective connections and reducing the bias. Our conservative decision resulted in a valuable first estimation moving forward in DoC research. Moreover, whole-brain models were built at a group level given a high amount of timepoints are required to simulate the probabilities of metastable substates. Future applications could benefit from other analytical approaches allowing for individualized patient brain models [17, 141, 142]. This way, accounting for transitions that do not hold if patients are no longer capable of supporting healthy dynamics due to the etiology and extent of brain damage [143]. In addition, we employed a parcellation with a limited number of nodes, which could hinder the specificity of substantial findings. Future work could benefit from higher-grained atlases, increasing the resolution of results. Finally, the classification of patients with DoC is an existing debate in neuroscience. Identifying MCS and UWS can depend on the CRS-R metric’s effectiveness, inter-rater variability, and consistency of caregivers’ reports [144]. It is challenging to distinguish between MCS and UWS since some patients classified as UWS may remain aware even though they do not demonstrate behavioral signs. They may be classified incorrectly as awake and unaware when they are actually conscious [145–148]. The circular nature of brain state definition and assessment could have compromised the efficacy and validity of our model definitions since they are subjected to the correct classification and typification of the empirical primary data source. It would be helpful to investigate the generalizability of our results with a broader range of DoC patients [3, 15].

## Conclusion

We were able to characterize and differentiate brain dynamics of DoC and healthy controls. We used a robust quantitative definition of brain states based on spontaneous spatiotemporal fluctuations [6, 28, 149]. Furthermore, we built whole-brain models to evaluate differences in reactivity to local perturbations. Crucially, our perturbation approach could be used as a specific model biomarker relating local activity with global brain dynamics. In light of the exciting results, causal whole-brain modeling can help understand other brain states and elucidate propagation properties, network level impact and sensitive areas when forcing transitions between brain states [142, 150, 151]. Overall, our results may eventually contribute to the field of artificial perturbation as a principled way of evaluating sensitivity in terms of perturbability, and understanding the underlying brain dynamics in DoC. Lastly, contemplating in the distant future clinical interventions with external perturbation to re-balance dynamics of post-coma patients towards more healthy regimes.

## Supporting information

S1 Fig Model-free results for all values of *k*. **a** Probability of occurrence for each group in each substate. Statistically significant differences are represented with asterisks (* p < 0.05, ** p < 0.01 and *** p < 0.001). Significant differences that did not survive correction by multiple comparisons are shown in black. **b** Leading eigenvectors of each substate. Blue corresponds to negative sign and red to positive sign.
(TIF)

S2 Fig Empirical analysis. Kuramoto order parameter calculated for each group. This measure captures phase coherence in a population of oscillators estimating the synchrony degree of the system. Higher values correspond to higher synchronization [79]. Less synchronous dynamics were revealed for MCS and UWS (in decreasing order) with respect to the control group. All comparisons had significant differences, represented with asterisks (* p < 0.05 and *** p < 0.001).
(TIF)

S3 Fig Empirical correlation analysis of total CRS-R. Correlation between the CRS-R of DoC with the probability of occurrence of each substate. There was no significant correlation in any of the substates.
(TIF)

S4 Fig Empirical correlation analysis of auditory, visual and motor CRS-R subscales. Correlation between 3 CRS-R subscales of DoC with the probability of occurrence of each substate: **a** Auditory, **b** Visual, **c** Motor. There was no significant correlation in any of the substates.
(TIF)

S5 Fig Empirical correlation analysis of verbal, communication and arousal CRS-R subscales. Correlation between 3 CRS-R subscales of DoC with the probability of occurrence of each substate: **a** Verbal, **b** Communication, **c** Arousal. There was no significant correlation in any of the substates.
(TIF)

S6 Fig Model-based analysis. **a** Evolution of KL distance as a function of *G* for MCS model. **b** Effective Connectivity of optimal fit for MCS model (*G* = 0.07). **c** Evolution of KL distance as a function of *G* for UWS model. **d** Effective Connectivity of optimal fit for UWS model (*G* = 0.04).
(TIF)

S7 Fig Perturbation correlation. Correlation between optimal transitions to the control state for MCS and UWS. Each point is the KL distance of the optimal stimulation of each brain area. The result is significant, showing regional patterns for MCS and UWS are spatially correlated.
(TIF)

S8 Fig Thalamic subcortical renders. Shen parcellation [66] node labels **a** 126, **b** 127, **c** 128, **d** 262, **e** 263 and **f** 264.
(TIF)

S9 Fig Perturbation sensitivity. Correlation between the KL distance of the optimal transition of each brain area, and the difference between healthy DTI and EC of **a** MCS and **b** UWS. Results are significant for both cases.
(TIF)

S10 Fig Perturbation statistics. We conducted perturbation analysis on three distinct brain areas, applying 30 iterations at their optimal stimulation intensity. The first area, located in the somatomotor network, exhibited the highest sensitivity for promoting the transition. The second was selected for being within the top 10% most sensitive and in the same network. The third was chosen as the mid-ranking node among the 214 nodes when ordered in descending order based on sensitivity. **a** Transition from MCS to to control state. Areas 160, 40 and 165 correspond to 56% left paracentral lobule and 34% left precental gyrus; 57% right rolandic operculum and 34% right insula; 81% left precentral gyrus, respectively. **b** Transition from UWS to control state. Areas 39, 26 and 229 correspond to 63% right postcentral gyrus; 51% right precentral gyrus and 43% right frontal superior; 42% left hippocampus and 5% left thalamus, respectively. All comparisons revealed significant differences, represented with asterisks (** p < 0.01 and *** p < 0.001).
(TIF)

S11 Fig Evolution of PMS during stimulation. **a** MCS Model. Evolution of PMS at different stimulation intensities for brain area 160 (56% left paracentral lobule and 34% left precental gyrus). Optimal fit to control state at a bifurcation value of *a* = 0.17. **b** UWS Model. Evolution of PMS at different stimulation intensities for brain area 39 (63% right postcentral gyrus). Optimal fit to MCS and control state at a bifurcation value of *a* = 0.13 and *a* = 0.15 respectively.
(TIF)

S1 Table Demographic and clinical information of DoC patients. Each row is a patient and each column corresponds to: patient characteristic (MCS or UWS), etiology (traumatic brain injury (TBI) and cerebral vascular accident (CVA)), time science injury (TSI), age, sex (0 = female, 1 = male), Coma Recovery Scale-Revised (CSR-R) auditory, visual, motor, verbal, communication and arousal subscores, CSR-R total.
(XLSX)

S2 Table Top 20 most sensitive regions for perturbing MCS model to control state. The first column corresponds to the KL distance between the PMS of the perturbed model, and the PMS of the target control state, after stimulating a given brain area. The second column shows the brain area in the Shen parcellation [66]. The third column indicates the overlap between the brain area and the AAL structural parcellation [80]. The rest of the columns are the X, Y and Z coordinates.
(XLSX)

S3 Table Top 20 most sensitive regions for perturbing UWS model to control state. The first column corresponds to the KL distance between the PMS of the perturbed model, and the PMS of the target control state, after stimulating a given brain area. The second column shows the brain area in the Shen parcellation [66]. The third column indicates the overlap between the brain area and the AAL structural parcellation [80]. The rest of the columns are the X, Y and Z coordinates.
(XLSX)

S4 Table Top 20 most sensitive regions for perturbing UWS model to MCS. The first column corresponds to the KL distance between the PMS of the perturbed model, and the PMS of the target control state, after stimulating a given brain area. The second column shows the brain area in the Shen parcellation [66]. The third column indicates the overlap between the brain area and the AAL structural parcellation [80]. The rest of the columns are the X, Y and Z coordinates.
(XLSX)

S5 Table Thalamus AAL parcellation. Node labels and coordinates following Shen parcellation [66] and their overlap with the AAL structural parcellation [80]. Abbreviations: L—left hemisphere; R—right hemisphere.
(XLSX)

S6 Table Thalamus Oxford Thalamic Connectivity Atlas parcellation. Node labels and their overlap with the Oxford Thalamic Connectivity Atlas [75]. This is an atlas for thalamocortical structural connectivity based on the structural connectivity of the thalamus with cortical areas (i.e., primary motor, primary somatomotor, occipital, premotor, prefrontal, parietal and temporal cortices). It is based on diffusion tractography in multiple subjects and reports the probability of the dominant connection. This is calculated as the fraction of subjects with probabilities higher than 0.25 to each of the 7 cortical zones, therefore the sum over all cortical areas should not sum to 1.
(XLSX)
