# Supplementary figures and images for: Re-awakening the brain: Forcing transitions in disorders of consciousness by external in silico perturbation
Source: PLoS Comput Biol. 2024 May 3;20(5):e1011350. doi: 10.1371/journal.pcbi.1011350 (PMC11068192; doi:10.1371/journal.pcbi.1011350)

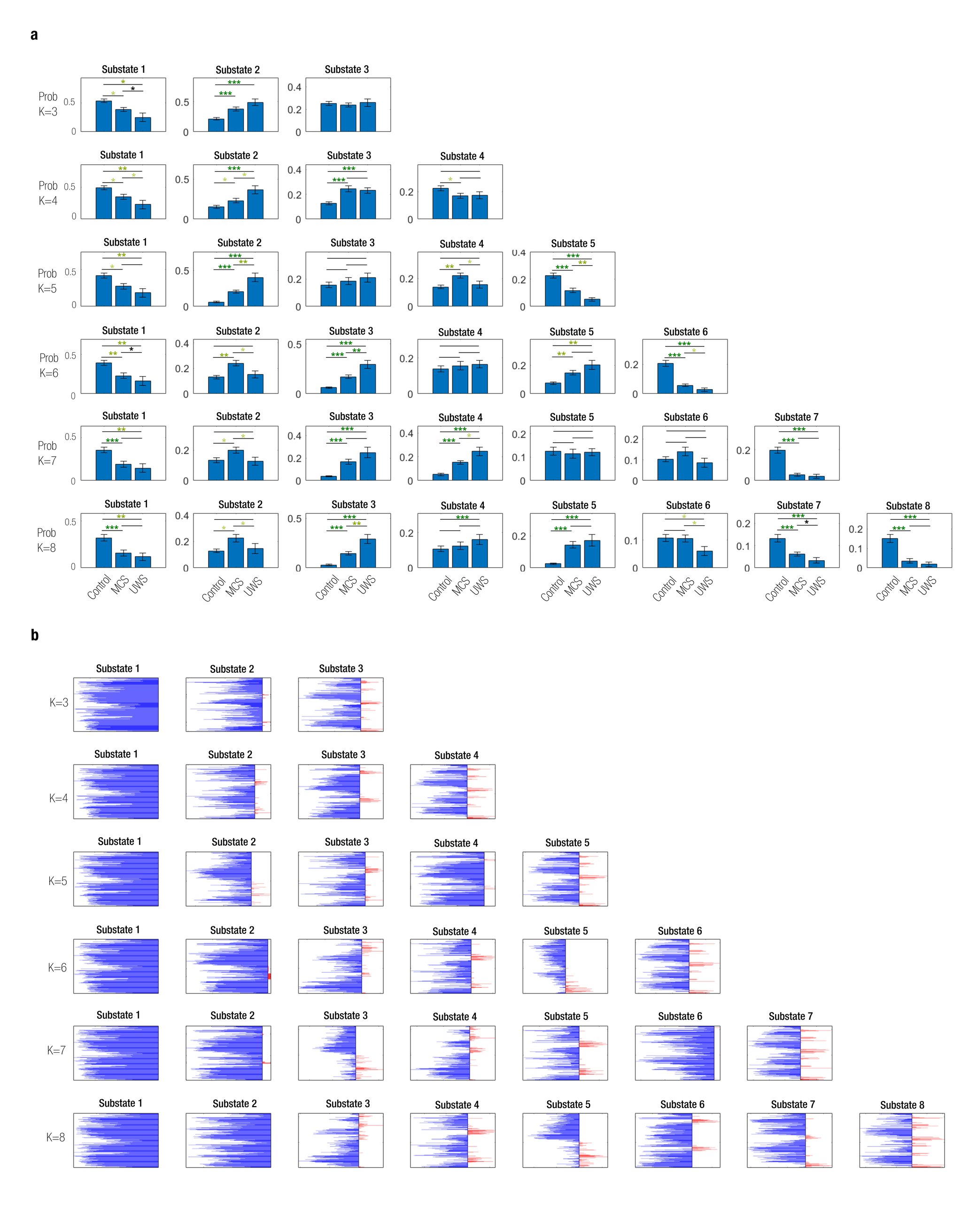

Supplement: S1 Fig — a Probability of occurrence for each group in each substate. Statistically significant differences are represented with asterisks (* p < 0.05, ** p < 0.01 and *** p < 0.001). Significant differences that did not survive correction by multiple comparisons are shown in black. b Leading eigenvectors of each substate. Blue corresponds to negative sign and red to positive sign. (TIF) [file pcbi.1011350.s001.tif]

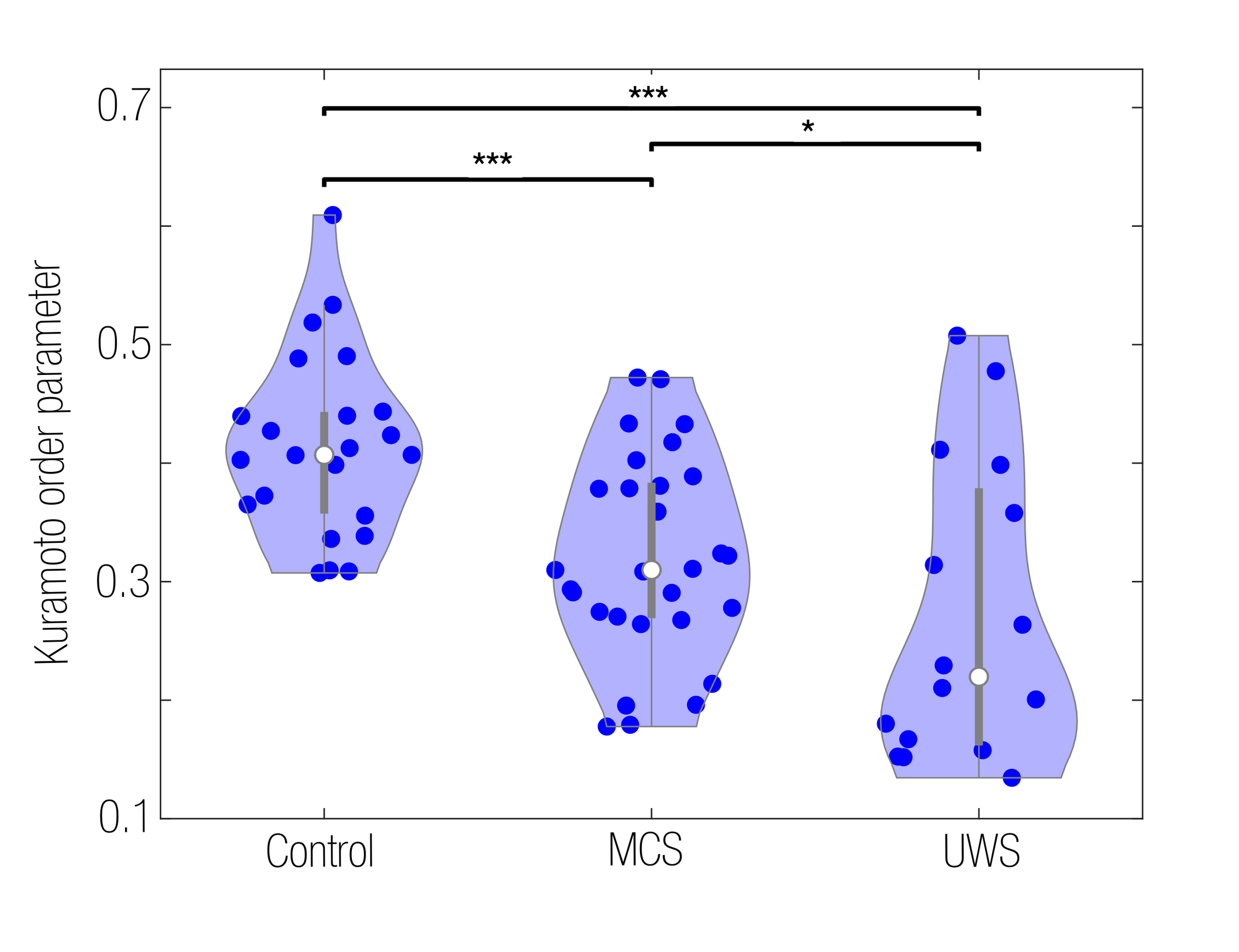

Supplement: S2 Fig — Kuramoto order parameter calculated for each group. This measure captures phase coherence in a population of oscillators estimating the synchrony degree of the system. Higher values correspond to higher synchronization [79]. Less synchronous dynamics were revealed for MCS and UWS (in decreasing order) with respect to the control group. All comparisons had significant differences, represented with asterisks (* p < 0.05 and *** p < 0.001). (TIF) [file pcbi.1011350.s002.tif]

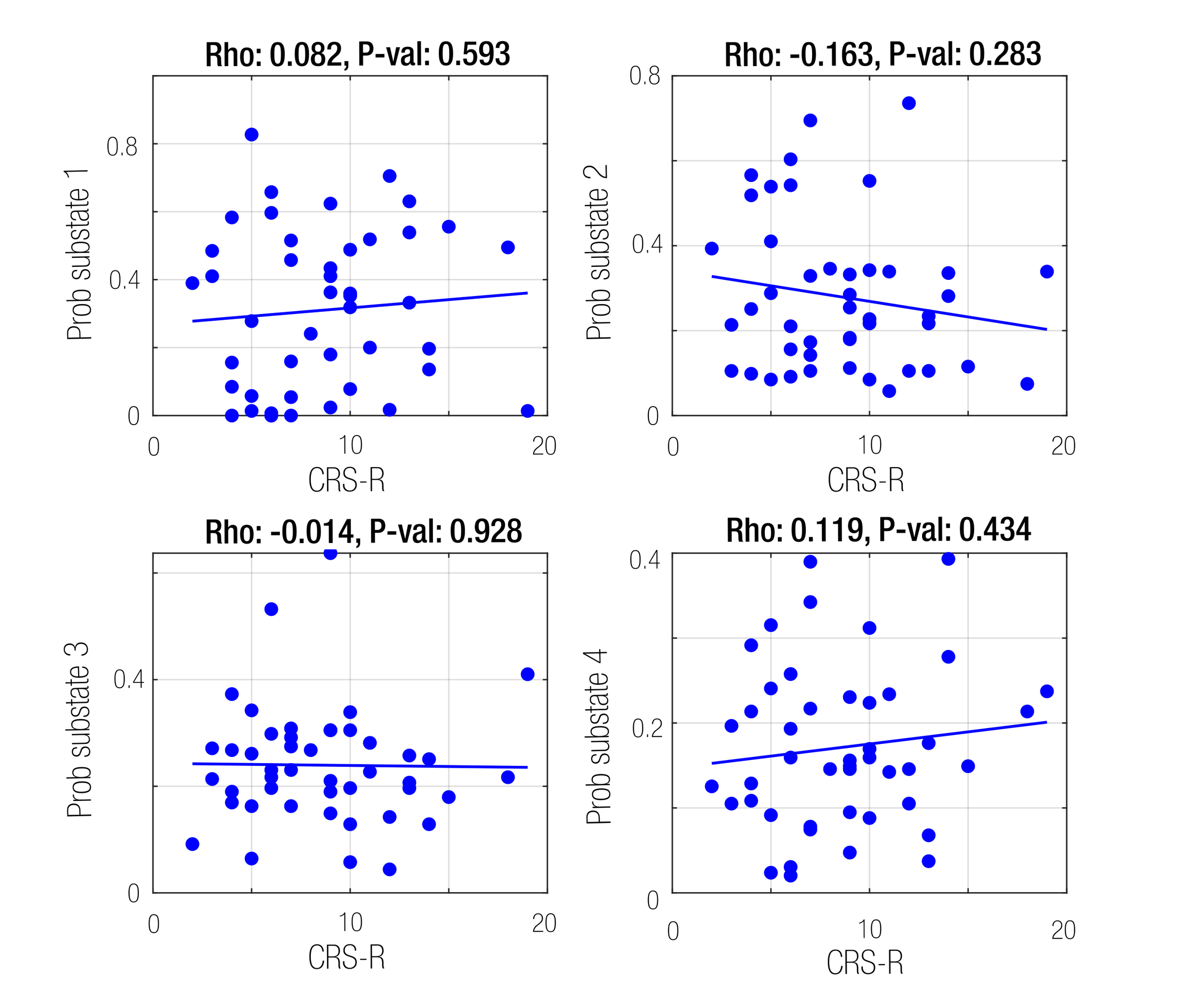

Supplement: S3 Fig — Correlation between the CRS-R of DoC with the probability of occurrence of each substate. There was no significant correlation in any of the substates. (TIF) [file pcbi.1011350.s003.tif]

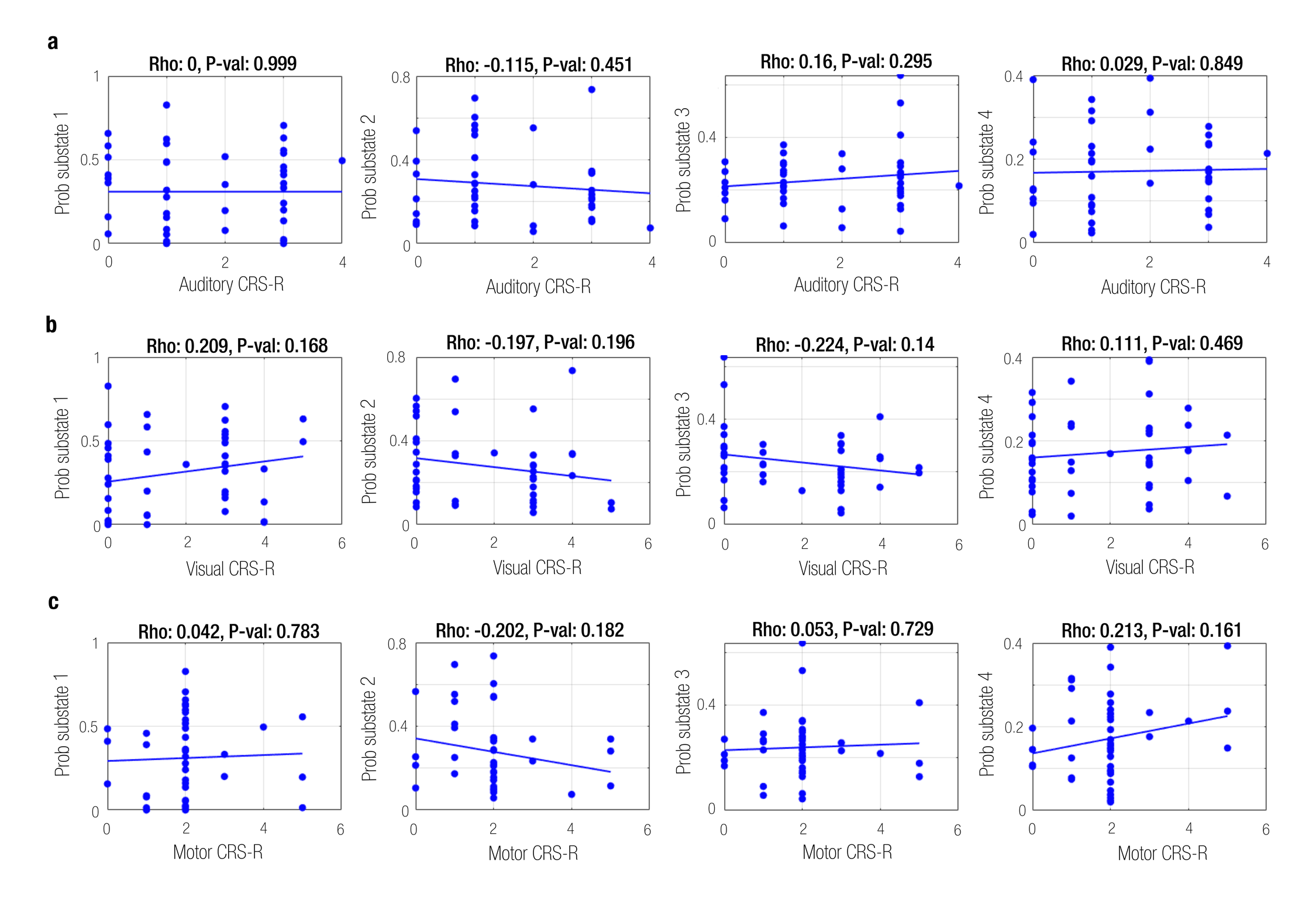

Supplement: S4 Fig — Correlation between 3 CRS-R subscales of DoC with the probability of occurrence of each substate: a Auditory, b Visual, c Motor. There was no significant correlation in any of the substates. (TIF) [file pcbi.1011350.s004.tif]

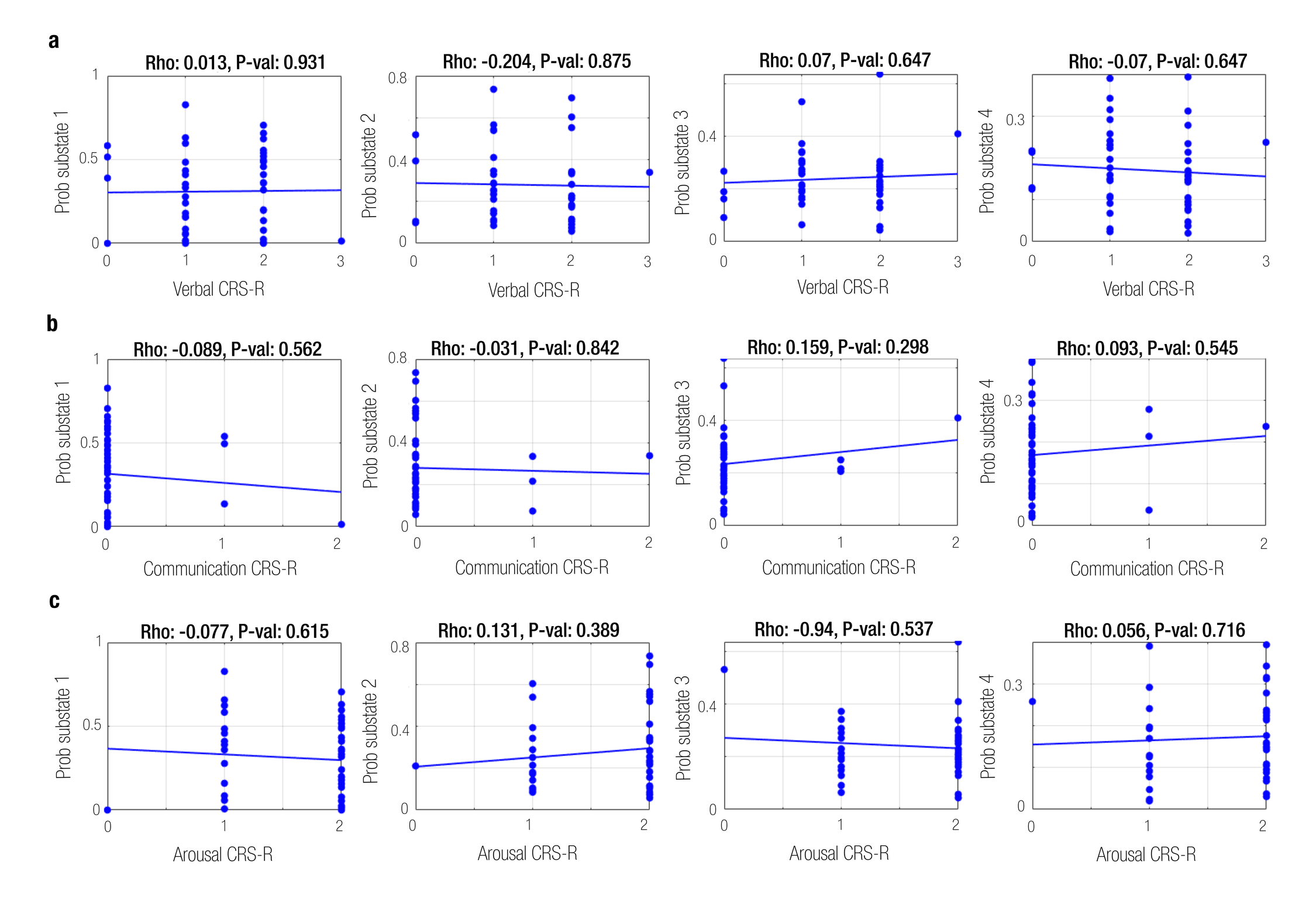

Supplement: S5 Fig — Correlation between 3 CRS-R subscales of DoC with the probability of occurrence of each substate: a Verbal, b Communication, c Arousal. There was no significant correlation in any of the substates. (TIF) [file pcbi.1011350.s005.tif]

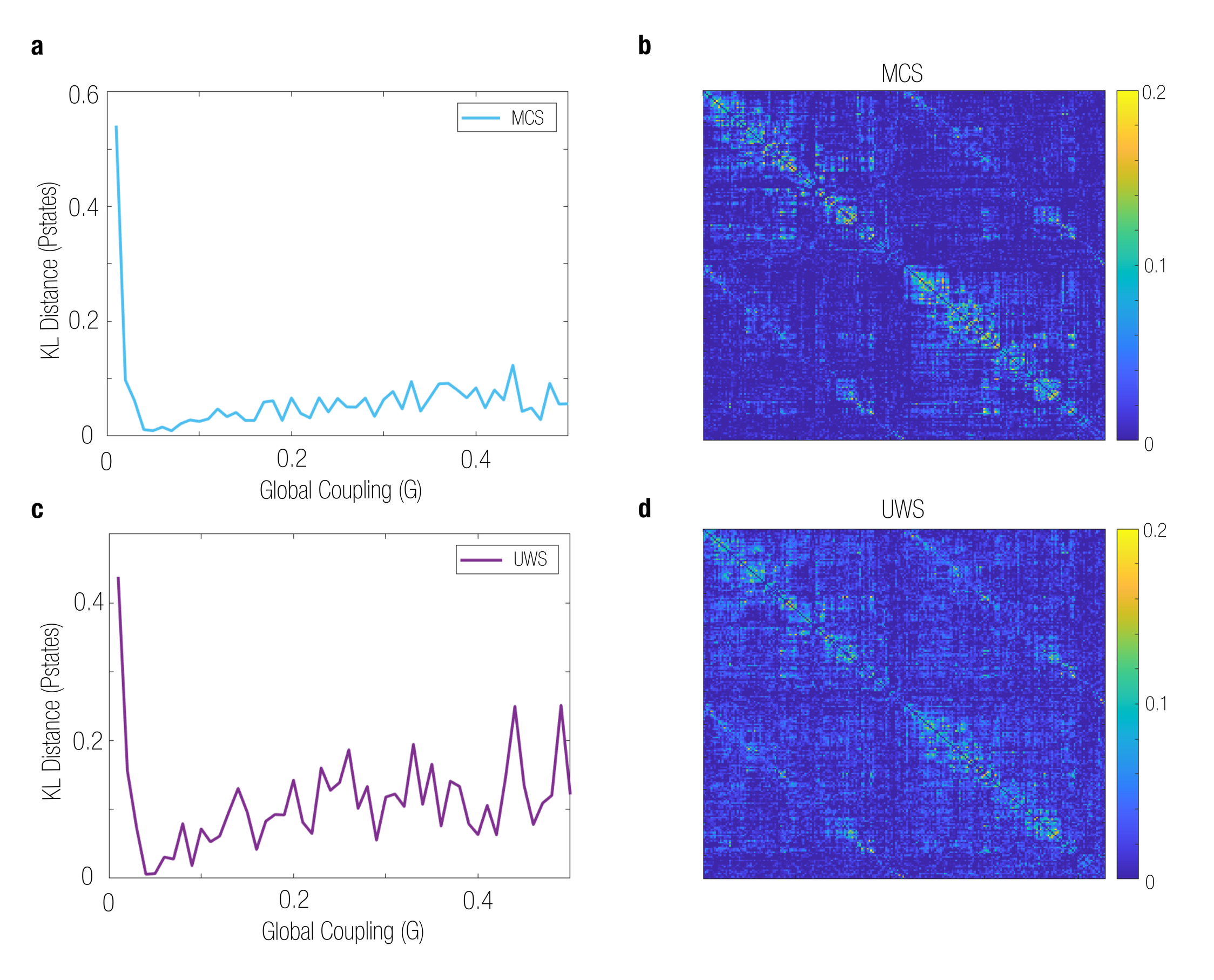

Supplement: S6 Fig — a Evolution of KL distance as a function of G for MCS model. b Effective Connectivity of optimal fit for MCS model (G = 0.07). c Evolution of KL distance as a function of G for UWS model. d Effective Connectivity of optimal fit for UWS model (G = 0.04). (TIF) [file pcbi.1011350.s006.tif]

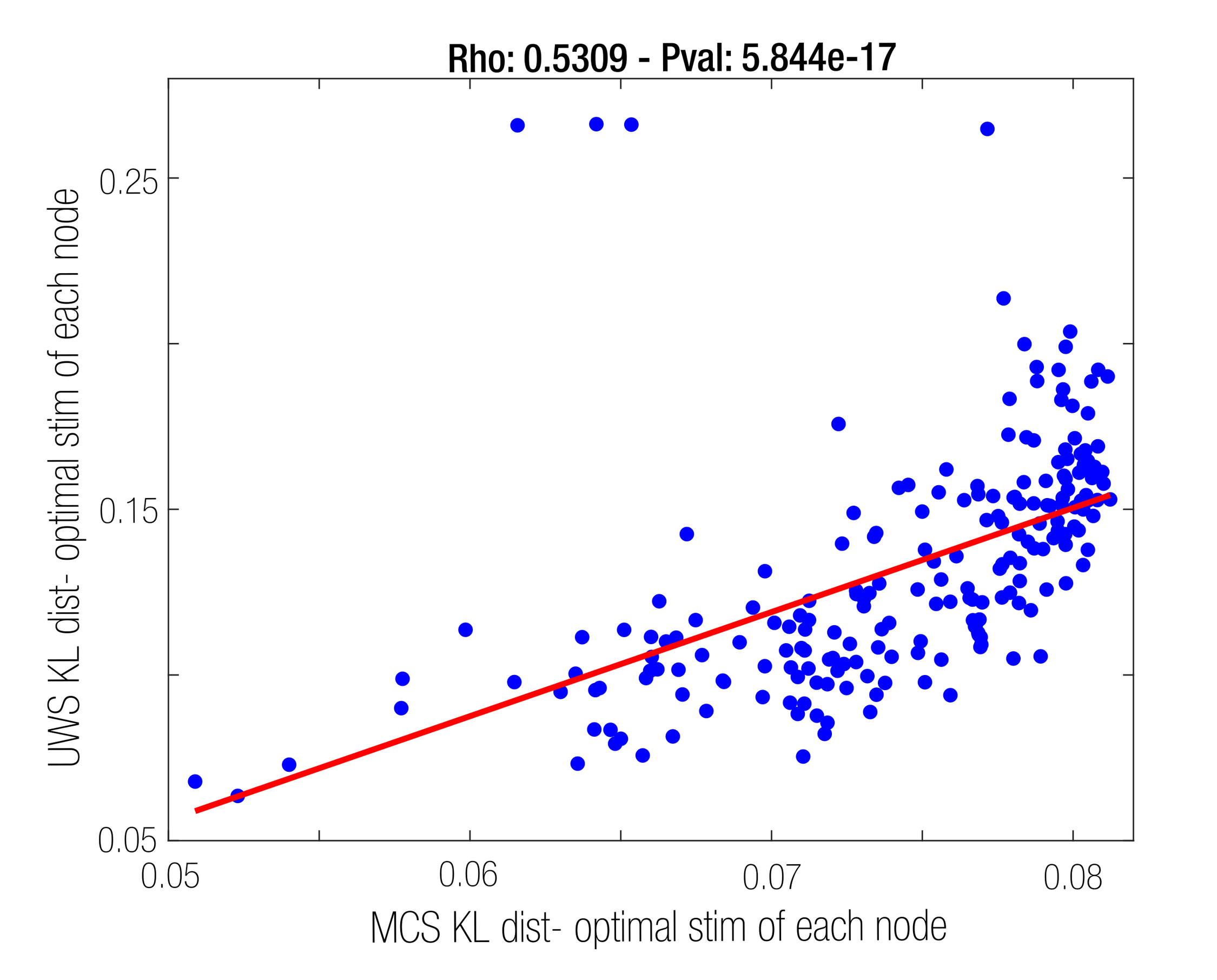

Supplement: S7 Fig — Correlation between optimal transitions to the control state for MCS and UWS. Each point is the KL distance of the optimal stimulation of each brain area. The result is significant, showing regional patterns for MCS and UWS are spatially correlated. (TIF) [file pcbi.1011350.s007.tif]

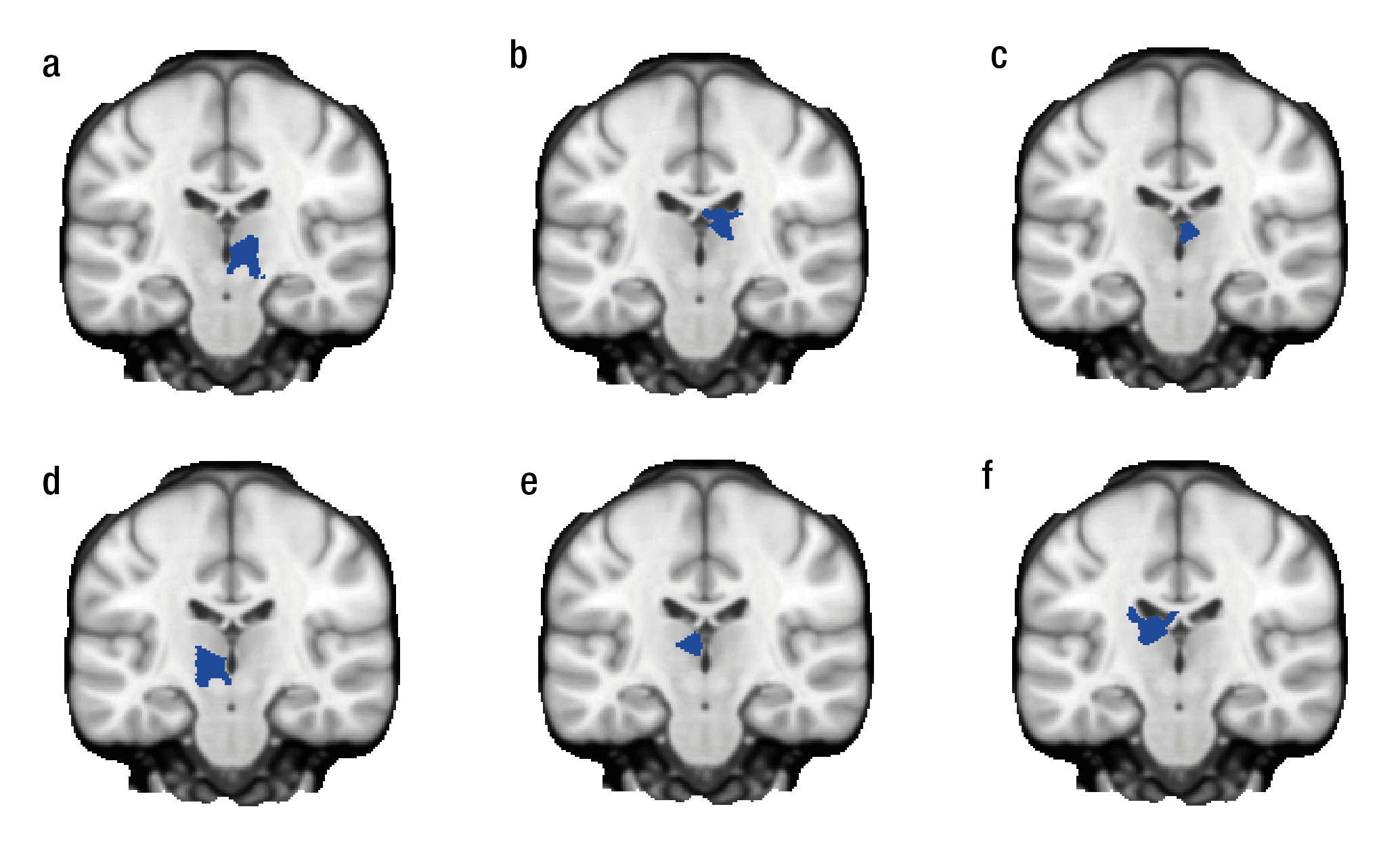

Supplement: S8 Fig — Shen parcellation [66] node labels a 126, b 127, c 128, d 262, e 263 and f 264. (TIF) [file pcbi.1011350.s008.tif]

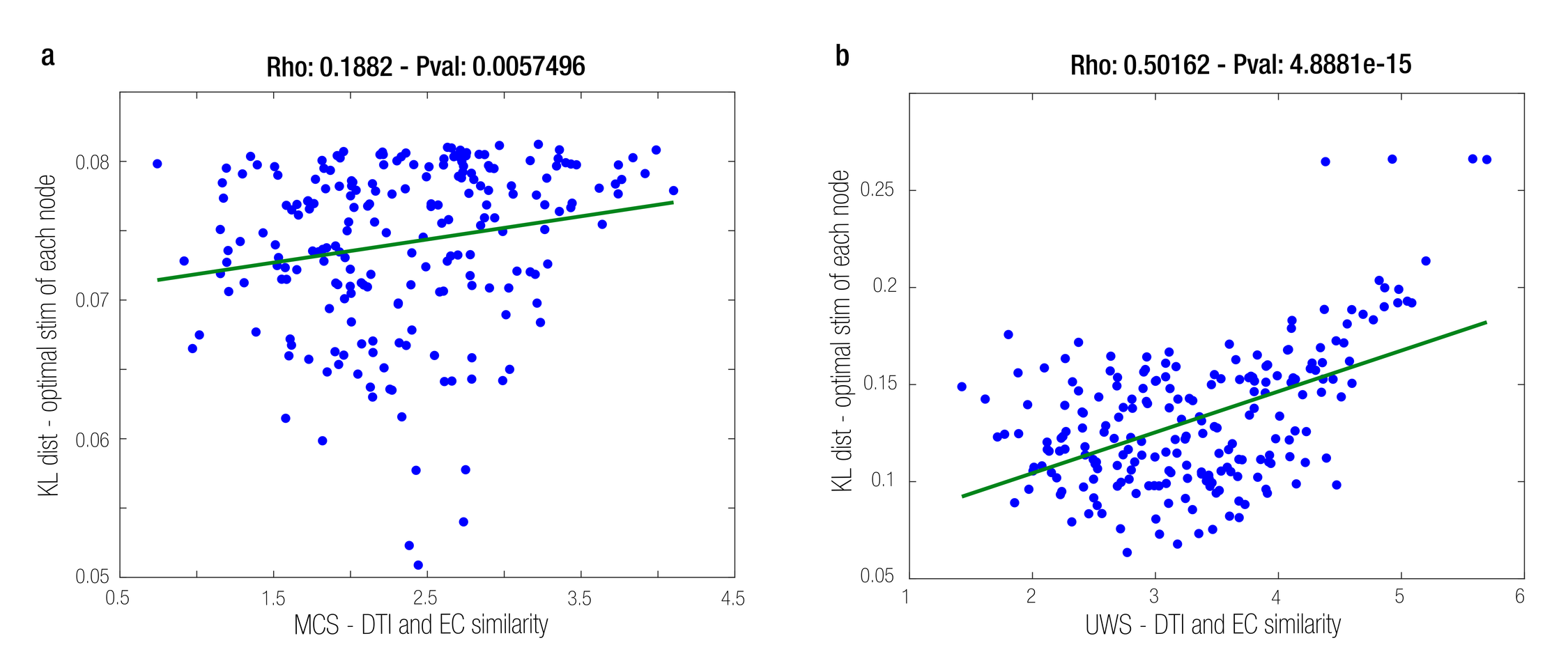

Supplement: S9 Fig — Correlation between the KL distance of the optimal transition of each brain area, and the difference between healthy DTI and EC of a MCS and b UWS. Results are significant for both cases. (TIF) [file pcbi.1011350.s009.tif]

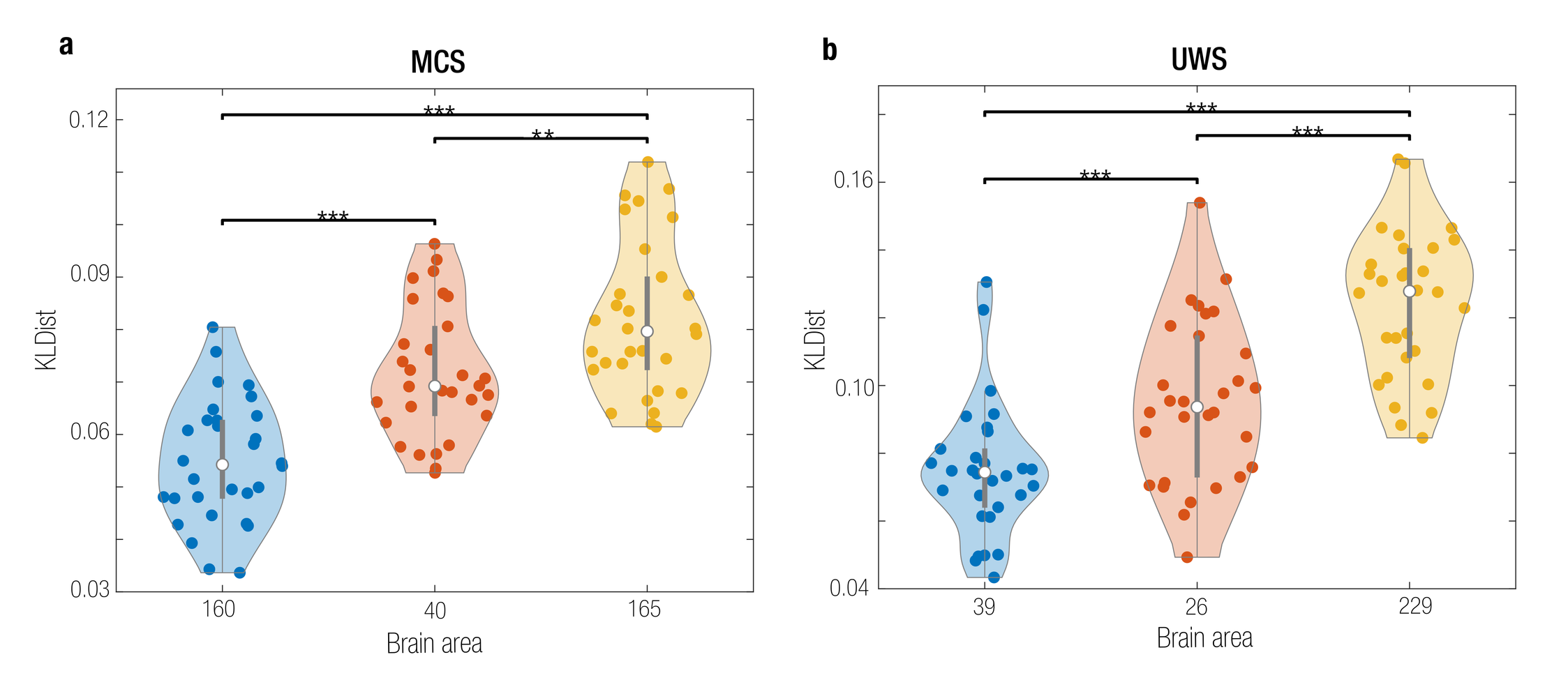

Supplement: S10 Fig — We conducted perturbation analysis on three distinct brain areas, applying 30 iterations at their optimal stimulation intensity. The first area, located in the somatomotor network, exhibited the highest sensitivity for promoting the transition. The second was selected for being within the top 10% most sensitive and in the same network. The third was chosen as the mid-ranking node among the 214 nodes when ordered in descending order based on sensitivity. a Transition from MCS to to control state. Areas 160, 40 and 165 correspond to 56% left paracentral lobule and 34% left precental gyrus; 57% right rolandic operculum and 34% right insula; 81% left precentral gyrus, respectively. b Transition from UWS to control state. Areas 39, 26 and 229 correspond to 63% right postcentral gyrus; 51% right precentral gyrus and 43% right frontal superior; 42% left hippocampus and 5% left thalamus, respectively. All comparisons revealed significant differences, represented with asterisks (** p < 0.01 and *** p < 0.001). (TIF) [file pcbi.1011350.s010.tif]

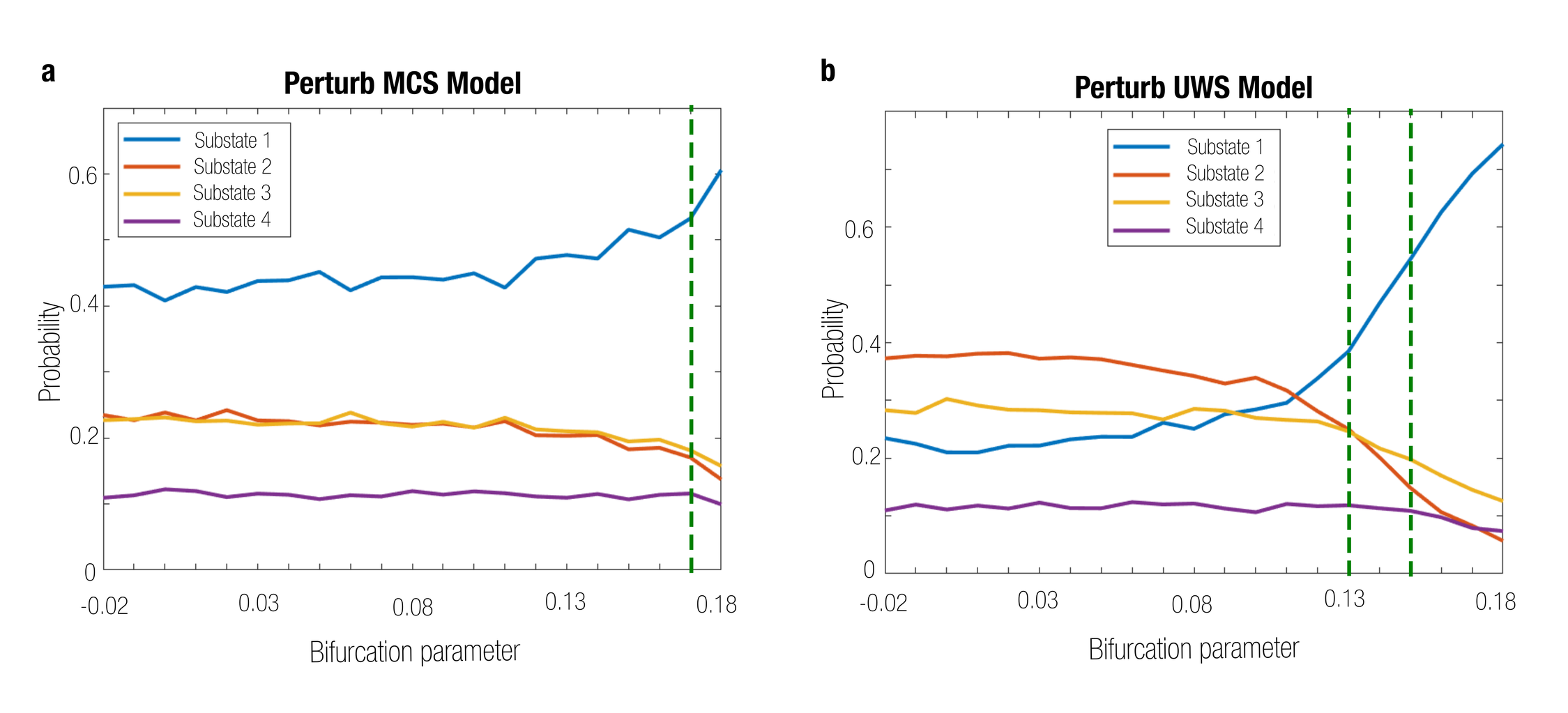

Supplement: S11 Fig — a MCS Model. Evolution of PMS at different stimulation intensities for brain area 160 (56% left paracentral lobule and 34% left precental gyrus). Optimal fit to control state at a bifurcation value of a = 0.17. b UWS Model. Evolution of PMS at different stimulation intensities for brain area 39 (63% right postcentral gyrus). Optimal fit to MCS and control state at a bifurcation value of a = 0.13 and a = 0.15 respectively. (TIF) [file pcbi.1011350.s011.tif]
